# p17/C18-ceramide–mediated mitophagy is an endogenous neuroprotective response in preclinical and clinical brain injury

**DOI:** 10.1093/pnasnexus/pgae018

**Published:** 2024-02-07

**Authors:** Eda Karakaya, Natalia Oleinik, Jazlyn Edwards, Jensen Tomberlin, Randy Bent Barker, Burak Berber, Maria Ericsson, Habeeb Alsudani, Adviye Ergul, Semir Beyaz, John J Lemasters, Besim Ogretmen, Onder Albayram

**Affiliations:** Department of Pathology and Laboratory Medicine, Medical University of South Carolina, Charleston, SC 29425, USA; Department of Biochemistry and Molecular Biology, Medical University of South Carolina, Charleston, SC 29425, USA; Hollings Cancer Center, Medical University of South Carolina, Charleston, SC 29425, USA; Department of Pathology and Laboratory Medicine, Medical University of South Carolina, Charleston, SC 29425, USA; Department of Pathology and Laboratory Medicine, Medical University of South Carolina, Charleston, SC 29425, USA; Department of Pathology and Laboratory Medicine, Medical University of South Carolina, Charleston, SC 29425, USA; Department of Pathology and Laboratory Medicine, Medical University of South Carolina, Charleston, SC 29425, USA; Department of Biology, Eskisehir Technical University, Tepebasi/Eskisehir 26555, Turkey; Electron Microscopy Laboratory, Department of Cell Biology, Harvard Medical School, Boston, MA 02115, USA; Cancer Center, Cold Spring Harbor Laboratory, Cold Spring Harbor, New York 11724, USA; College of Science, University of Basrah, Basra 61004, Iraq; Department of Pathology and Laboratory Medicine, Medical University of South Carolina, Charleston, SC 29425, USA; Ralph H. Jackson Department of Veterans Affairs Medical Center, Charleston, SC 29425, USA; Cancer Center, Cold Spring Harbor Laboratory, Cold Spring Harbor, New York 11724, USA; Department of Biochemistry and Molecular Biology, Medical University of South Carolina, Charleston, SC 29425, USA; Hollings Cancer Center, Medical University of South Carolina, Charleston, SC 29425, USA; Department of Drug Discovery and Biomedical Sciences, Medical University of South Carolina, Charleston, SC 29425, USA; Department of Biochemistry and Molecular Biology, Medical University of South Carolina, Charleston, SC 29425, USA; Hollings Cancer Center, Medical University of South Carolina, Charleston, SC 29425, USA; Department of Pathology and Laboratory Medicine, Medical University of South Carolina, Charleston, SC 29425, USA; Ralph H. Jackson Department of Veterans Affairs Medical Center, Charleston, SC 29425, USA; Department of Neuroscience, Medical University of South Carolina, Charleston, SC 29425, USA

## Abstract

Repeat concussions (or repetitive mild traumatic brain injury [rmTBI]) are complex pathological processes consisting of a primary insult and long-term secondary complications and are also a prerequisite for chronic traumatic encephalopathy (CTE). Recent evidence implies a significant role of autophagy-mediated dysfunctional mitochondrial clearance, mitophagy, in the cascade of secondary deleterious events resulting from TBI. C18-ceramide, a bioactive sphingolipid produced in response to cell stress and damage, and its synthesizing enzyme (CerS1) are precursors to selective stress-mediated mitophagy. A transporter, p17, mediates the trafficking of CerS1, induces C18-ceramide synthesis in the mitochondrial membrane, and acts as an elimination signal in cell survival. Whether p17-mediated mitophagy occurs in the brain and plays a causal role in mitochondrial quality control in secondary disease development after rmTBI are unknown. Using a novel repetitive less-than-mild TBI (rlmTBI) injury paradigm, ablation of mitochondrial p17/C18-ceramide trafficking in p17 knockout (KO) mice results in a loss of C18-ceramide–induced mitophagy, which contributes to susceptibility and recovery from long-term secondary complications associated with rlmTBI. Using a ceramide analog with lipid-selenium conjugate drug, LCL768 restored mitophagy and reduced long-term secondary complications, improving cognitive deficits in rlmTBI-induced p17KO mice. We obtained a significant reduction of p17 expression and a considerable decrease of CerS1 and C18-ceramide levels in cortical mitochondria of CTE human brains compared with age-matched control brains. These data demonstrated that p17/C18-ceramide trafficking is an endogenous neuroprotective mitochondrial stress response following rlmTBI, thus suggesting a novel prospective strategy to interrupt the CTE consequences of concussive TBI.

**Significance Statement**

Repeat concussions (or repetitive mild traumatic brain injury [rmTBI]) involve complex pathological processes consisting of primary insults and long-term secondary complications and are a prerequisite for chronic traumatic encephalopathy (CTE). Autophagy-mediated dysfunctional mitochondrial clearance, known as mitophagy, plays a significant role in the cascade of secondary deleterious events resulting from rmTBI. This study presents a novel finding, i.e. a new experimental model of CTE (repetitive less-than-mild closed head injury paradigm), which provides insights into how the brain becomes vulnerable to developing secondary sequelae after repetitive concussive traumatic brain injuries (TBIs). The findings derived from preclinical and clinical data suggest that a novel mitophagy pathway is an endogenous neuroprotective response following rmTBI, thus, a novel therapeutic target for interrupting the CTE consequences of concussive TBIs.

## Introduction

Traumatic brain injury (TBI) is a prerequisite for chronic traumatic encephalopathy (CTE) (1, 2), as well as a major environmental risk factor for Alzheimer's disease–related dementia (3, 4). Although the initial damage is irreversible in TBI, secondary insults contribute to long-term sequelae in a reversible manner (5). Therefore, determining how secondary insults develop from the initial injury and identifying therapeutic targets are crucial for managing the symptoms and preventing the long-term sequelae of TBI. The multitude of mechanisms and pathways in the secondary injury cascade of TBI offers numerous prospective mechanisms for neuronal protection (6, 7). Of these, mitochondria, the major subcellular components essential for proper cellular functioning and cell survival, are of particular interest since recent studies have strongly implied that mitochondrial dysregulation is a major driver of the secondary deleterious cascades following brain injuries (8–14). The maintenance of mitochondrial dynamics is a necessity for maintaining the integrity of the mitochondrial pool for neuronal homeostasis, which, in turn, impacts various physiological and pathological pathways in the brain (15–18). Therefore, adjustment of the imbalance in mitochondrial dynamics may exert beneficial effects on mitochondrial structure and function, as well as increase axonal/neuronal survival after TBI. However, the mechanism by which mitochondrial dynamics affect mitochondrial health and axonal/neuronal survival is a relatively new and active area of TBI research.

The molecular mechanisms for the removal of dysfunctional mitochondria and regulating the processes of dynamic mitochondrion pool are thought of as crucial in neuronal health and survival. Maintenance of the mitochondrion pool requires both fusion and fission to properly identify and remove damaged organelles (19). Mitochondrial fusion allows for greater energy production during conditions of high metabolic activity, whereas fission facilitates the transport of mitochondria to high-energy regions and the removal of damaged mitochondria via mitophagy (19). C18-ceramide (C18-Cer) is produced in response to cell stress and plays a vital role in stress-mediated mitophagy (20–23). The subcellular localization of CerS1 by the novel p17/PERMIT protein (17 kDa transporter) in damaged mitochondria vs. endoplasmic reticulum (ER) to induce C18-Cer generation, and the subsequent stress requires LC3 (microtubule-associated light chain protein 3) activation and mitophagy in various metabolically active tissues (24), including the brain (25). However, the causal role of p17/C18-Cer–associated mitochondrial quality control in the brain and its mitochondrial signaling in repetitive concussive TBI and its clinical relevance remain completely unknown. To address the knowledge gap, we implemented a novel repetitive less-than-mild TBI (rlmTBI) paradigm in young and old mice to analyze the age-dependent lasting neurological effects and cognitive outcomes. Then, we uncovered that p17-mediated mitophagy is an endogenous neuroprotective response, which provides new mechanism-based therapeutic options to attenuate secondary disease progression after concussive TBI, which underlies CTE development.

## Results

### Age-at-injury determines the extent of long-term neuropathology and cognitive outcomes in novel repetitive less-than-mild closed head injury model

The existing models of repetitive mild TBIs (rmTBIs) are capable of replicating most of the histopathological and functional outcomes that are observed in the clinical features of CTE. However, these models do not provide an explanation for why the development of secondary diseases may not necessarily progress or be specific to individuals who have suffered from repetitive brain trauma. The exact prevalence of CTE is still unknown, and despite extensive research, the factors that cause resistance and resilience to neuropathology and clinical disease have not yet been definitively identified. To investigate this phenomenon, we have first developed a new experimental model of repetitive concussive brain injury, known as the rlmTBI paradigm, based on our and others’ previous reports (26–30).

As part of a proof-of-concept experiment, 2-month-old male C57BL/6 wild-type (WT) mice were subjected to seven *mild* (54-g weight from 36″ drop height yields an impact energy of 0.484 J) OR *less-than-mild* (54-g weight from 24″ drop height yields an impact energy of 0.323 joules) hits to the dorsal aspect of the skull over 9 days (Fig. S1A). The impact energy was obtained by multiplying the mass of the weight (kg) with the gravity force (9.8 m/s^2^) and the height from where it is launched (0.91 or 0.60 m, respectively), as previously reported (31). The loss of the righting reflex (loss of consciousness) was evaluated based on the latency of self-righting immediately after the injuries, which correlates with injury severity (32). The average time elapsed before recovery of the righting reflex was significantly increased in both rmTBI (*P* < 0.0001) and rlmTBI (*P* < 0.0001) mice, compared with the sham controls (Fig. S1B). However, we observed that mice subjected to rmTBI took a significantly longer time to self-right than those exposed to rlmTBI during the first 3 days (*P* = 0.01; Fig. S1B). In good accordance with our previous reports (26, 27, 29), at 8 months postinjury, the existing rmTBI model induced significant long-term neurological and cognitive deficits (Fig. S1C and D) accompanied by cortical progressive degeneration of white matter (WM; Fig. S1E and F), axonal pathology (Fig. S2A), and tau pathology (Figs. S2B, S2C, and S3A–F). In contrast, receiving rlmTBI at 2 months of age did not cause any long-term impairments.

**Fig. 1.**
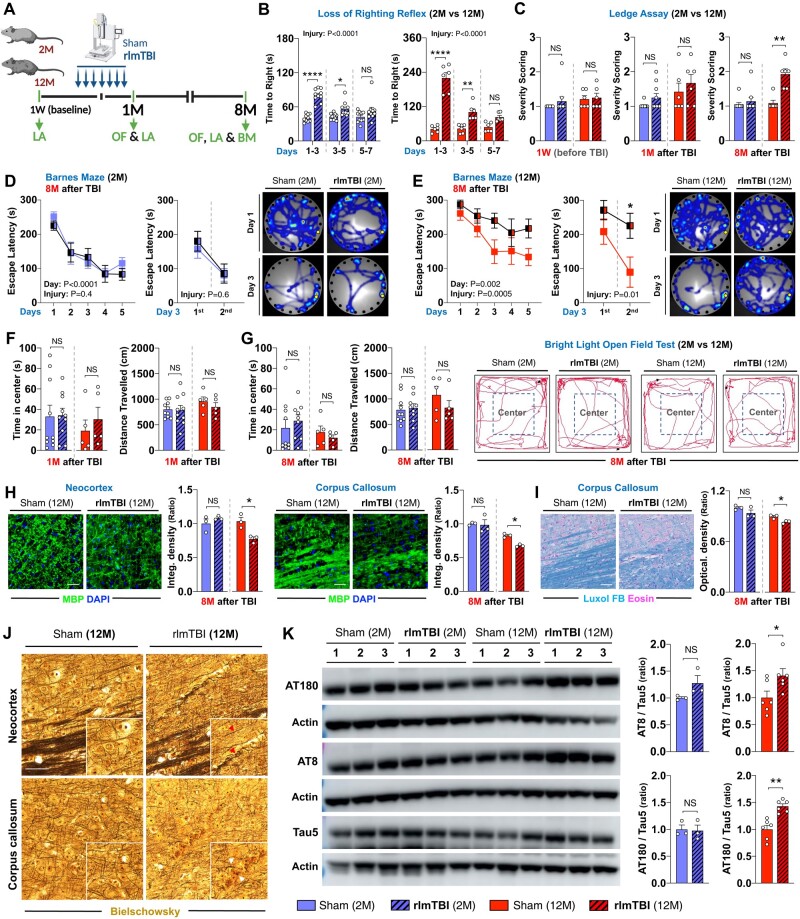
The mouse model of new repetitive mild closed head injury demonstrates age-at-injury–dependent secondary neuropathological changes and cognitive impairments. A) Experimental setup. Two-month-old (2M) and 12-month-old (12M) male C57BL/6J WT mice underwent rlmTBIs or sham injuries (Created with Biorender.com), and (B) the latency of their righting reflex was recorded, followed by functional and pathological examination for 8 months. The long-term effects of the novel rlmTBI on neurological deficits in both the 2M and 12M mice groups were longitudinally assessed by (C) the Ledge assay (LA) in mice before rlmTBI and 1 and 8 months after the last injury. Cognitive performance was assessed by Barnes maze (BM) in 2M (D) and 12M (E) mice; escape latency across the 5 days of acquisition phase (left) and two consecutive sessions (right, 5 min interval) on day 3 of the acquisition phase; and movement heat map on days 1 and 3 (the occupancy rate is graded by a color map ranging from cold to warm colors). F and G) Anxiety-like behavior was assessed by bright light, open-field (OF) in both groups; time spent in the center (left) and distance traveled (right) and trajectory plots at 1 and 8 months after the last injury. The long-term neuropathological consequences of rlmTBI of the 2M and 12M mice groups on the axonal degeneration (axonopathy) and tau pathology, as shown by immunofluorescence (IF) for MBP (H) and LFB staining (I) to myelinated axonopathy and Bielschowsky silver staining (J) to disruption of axons and the presence of axonal swellings and spheroids (red arrow heads indicate axonal swellings; white arrow heads indicate spheroids) and immunoblotting for AT180, AT8, and Tau5 (total tau) (K) to abnormal tau phosphorylation in neocortex and corpus callosum. Inset images are the high magnification images of the selected area denoted by white. Scale bar, 50 μm. Data are expressed as mean ± SEM (two-way ANOVA with Bonferroni's correction and unpaired two-tailed nonparametric t test). NS, not significant. *P < 0.05, **P < 0.01, ***P < 0.001, and ****P < 0.0001.

The aged brain is particularly vulnerable to secondary disease development after TBI, making it more susceptible to chronic neurodegenerative changes following TBIs (33–36). To test whether age-at-injury hinders long-term recovery in a new rlmTBI paradigm, young (2-month-old) and old (12-month-old) male C57BL/6 WT mice were subjected to 7 less-than-mild hits to the dorsal aspect of the skull in 9 days (Fig. 1A). Long-term neurological and cognitive performance, WM and axonal degeneration, and development of tau pathologies were assessed 8 months post-rlmTBI in an age-dependent manner. The average time elapsed before recovery of the righting reflex was significantly increased in both 2-month-old (*P* < 0.0001) and 12-month-old (*P* < 0.0001) rlmTBI mice, compared with their age-matched sham controls (Fig. 1B). Then, long-term neurological and cognitive effects of rlmTBI were assessed using a Ledge assay and Barnes maze, respectively, 8 months after the last injury. Although rlmTBI-induced 2-month-old and all sham group mice did not demonstrate long-term neurological impairments, rlmTBI-induced 12-month-old mice developed significant neurological deficits at 8 months postinjury, analyzed by Ledge assay (Fig. 1C). At 8 months after the last injury, two-way ANOVA also revealed that rlmTBI-induced 12-month-old (*P* = 0.001), but not 2-month-old (*P* = 0.4), mice showed a significantly increased escape latency to find the escape hole compared with their age-matched sham controls in the Barnes maze test (Figs. 1E and D and S3A and B). We did not observe any significant changes in anxiety-like behavior in both 2-month-old and 12-month-old rlmTBI mice (1 and 8 months after the last injury) compared with their age-matched sham controls, evaluated by the bright light, open-field test (Fig. 1F and G).

The biomechanical forces involved in repetitive concussive brain injuries lead to diffuse axonal injury because WM tracts are particularly susceptible to mechanical loading (37). Thus, we investigated WM and axonal integrity in our mice 8 months after the injuries. The microstructure of WM components was assessed by immunostaining for myelin basic protein (MBP), a marker for myelin, and by Luxol Fast Blue (LFB) staining. Our results demonstrated that progression of WM pathology in 12-month-old, but not 2-month-old, mice (8 months after the last injury) after rlmTBI with traumatic axonal injury was localized in the neocortex and corpus callosum, as identified by a significant decrease in the analysis of the integrated density of MBP (Fig. 1H) and LFB (Fig. 1I), in the absence of overt neuronal loss (data not shown). Notably, Bielschowsky silver staining revealed that the neocortex and corpus callosum contained oriented and extended bundles of axons and large axonal swellings, which may have developed due to WM reorganization in 12-month-old, but not 2-month-old, mice (8 months after the last injury) in rlmTBI (Fig. 1J). Tau protein assembles axonal microtubule bundles, which are important structural elements in the axonal cytoskeleton (38). Previous results from experimental studies in animals suggest that intra-axonal hyperphosphorylated tau (P-tau) accumulation may be consequences of repeated brain trauma (39). However, the link between P-tau and TBI in rodents remains controversial, and the extent to which potential genetic and environmental factors contribute to tau pathology is unknown. Thus, using the aged brain paradigm, we assessed the long-term effects of novel rlmTBI on the development of early-stage neurofibrillary-like pathology in the neocortex using AT8 and AT180, which recognizes P-tau epitopes at Ser202/Thr205 and Thr231 residues, respectively. Notably, we observed that although total tau (tau-5) levels remained unchanged, rlmTBI increased phospho-tau AT8 and AT180 in the neocortex of 12-month-old, but not the 2-month-old, mice at 8 months postinjury (Figs. 1K and S4C–E).

### p17-associated mitophagic response contributes to the susceptibility to and recovery from secondary injuries post-rlmTBI

**Fig. 2.**
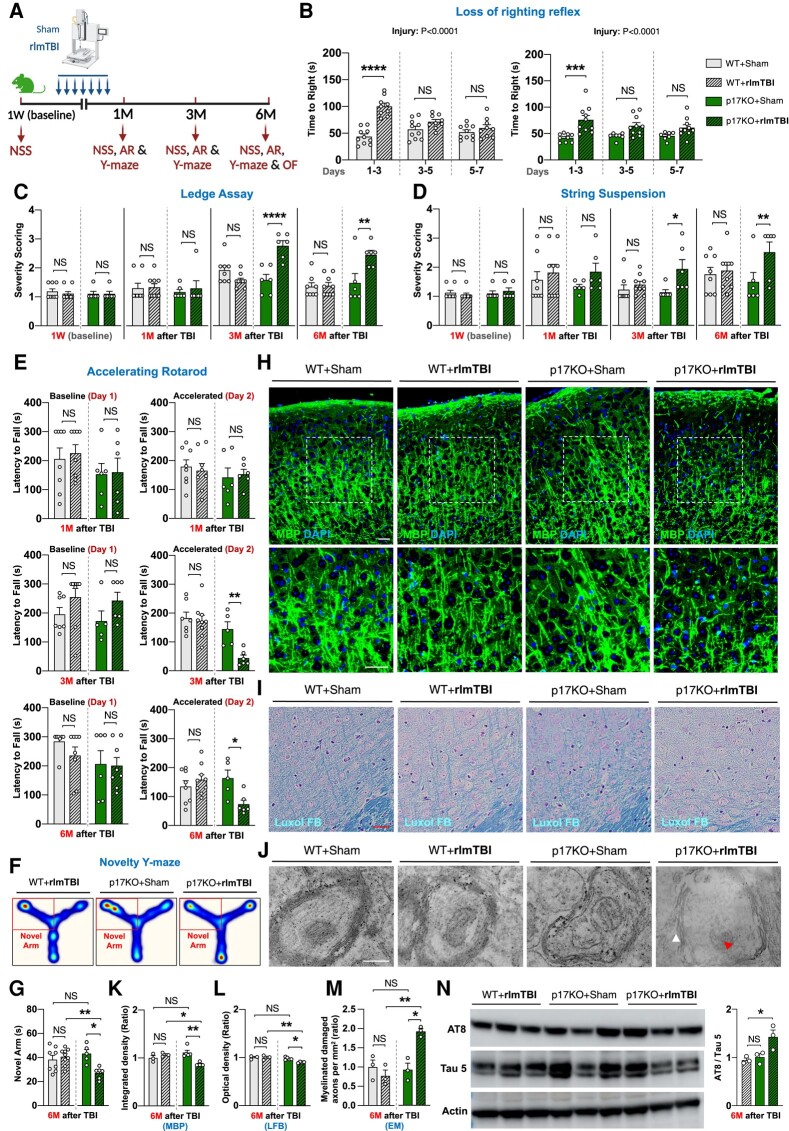
Ablation of p17 affects the long-term functional recovery and development of secondary axonal degeneration after rlmTBI. A) Experimental setup. Two-month-old male p17KO and C57BL/6J WT mice underwent rlmTBIs or sham injuries, and (B) the latency of their righting reflex was recorded, followed by functional and pathological examination for 6 months. Their neurological scoring (NSS) was longitudinally assessed by (C) Ledge assay and (D) string suspension test in mice before TBI and 1, 3, and 6 months after the last injury. E) Sensorimotor competency was longitudinally assessed with the accelerated rotarod (AR) in mice 1, 3, and 6 months after the last injury (day 1 represents baseline performance; maximum time is 300 s). Working memory and cognitive flexibility were assessed by novelty Y-maze (F), movement heat map (the occupancy rate is graded by a color map ranging from cold to warm colors), and (G) cumulative time spent in novel arm on test phase. The microstructural organization of myelinated axons and axonal integrity were assessed by immunostaining for (H) MBP, (I) LFB myelin staining, and (J) electron microscopy (red arrow head indicate degenerated axonal mitochondria; white arrow head indicate disorganized myelin attachment to axons at paranodes; scale bar, 500 nm) in the neocortex 6 months after the last injury. Inset images are high magnifications of representative areas. Bar graph showing the quantification of integrated fluorescence density of (K) MBP immunostaining and optical density of (L) LFB staining in the neocortex of mice in each group (*n* = 3–5). Scale bars, 50 μm. M) Bar graph showing the quantification of the relative myelinated damaged axons at electron microscopy (EM) imagining in the neocortex of mice in each group (*n* = 3). N) Western blot analysis of cortical tau pathology was analyzed for AT8, AT100, and Tau5 (total tau) at 6 months after rlmTBIs (*n* = 3). Data are expressed as mean ± SEM (two-way ANOVA with Bonferroni's correction and one-way ANOVA with Dunnett's correction). NS, not significant. *P < 0.05, **P < 0.01, ***P < 0.001, and ****P < 0.0001.

Mitochondria play a significant role in ensuring proper neuronal axon functioning and survival, and recent studies have strongly implicated mitochondrial dysregulation as a major driver of the secondary damage to axons, which occurs following brain injury (40, 41). Notably, our recent study showed that the mitochondrial trafficking of CerS1 by the novel p17 transporter induces C18-Cer synthesis and accumulation in mitochondria undergoing mitophagy in various metabolically active tissues (24). Mitochondrial C18-Cer then acts as a receptor for LC3-containing autophagosomes for the recruitment and removal of damaged mitochondria by ceramide-dependent mitophagy (24). To study the impact of deleting p17 on the long-term recovery and development of secondary axonal degeneration in the brain in both sexes after rlmTBI, 2-month-old male and female p17KO and C57BL/6 WT mice were subjected to seven less-than-mild injuries to the dorsal aspect of the skull or sham injury over a period of 9 days. The loss of the righting reflex was immediately assessed after each injury, while baseline and postinjury behavioral testing were assessed longitudinally at 1, 3, and 6 months following rlmTBI (Fig. 2A). The average time elapsed before recovery of the righting reflex was significantly increased in WT (*P* < 0.0001 for both males and females) and p17KO (*P* < 0.0001 for both males and females) rlmTBI mice compared with their sex-matched sham controls (Figs. 2B and S6B). No sex-injury interaction was found for average righting reflex time (*P* = 0.9 for both p17KO and WT mice), suggesting injury severity was similar between males and females. Then, we longitudinally assessed long-term neurological and cognitive deficits after rlmTBI in both sexes. Although rlmTBI-induced WT nor WT or p17KO sham male mice did not demonstrate behavioral impairments, rlmTBI-induced p17KO male mice developed significant sensorimotor deficits in the secondary injury phase starting at 3 months postinjury, analyzed by Ledge assay (Fig. 2C), string suspension (Fig. 2D), and accelerated rotarod tests (Fig. 2E). Additionally, we conducted subsequent novelty Y-maze tests at 1, 3, and 6 months postinjury to longitudinally monitor cognitive impairment following rlmTBI. The results revealed that 6 months after the last injury, cognitive deficits appeared (Figs. 2F and G and S5A–C), indicating a slowly developing cascade of long-term cognitive deficits in rlmTBI-induced p17KO male mice. Notably, opposing effects of p17 depletion were confirmed through behavioral assays, demonstrating a significant long-term secondary behavioral impairment in TBI-induced p17KO males, while rlmTBI-induced p17KO females were protected from secondary behavioral deficits (Fig. S6A–E). We did not observe any significant changes in anxiety-like behavior and hyperactivity at 6 months after the last injury in both male (Fig. S5D and E) and female (Fig. S7A) p17KO and WT rlmTBI mice compared with their sex-matched and age-matched sham controls, evaluated by the bright light, open-field test.

WM tracts traversing the brain contain long myelinated axons that are especially vulnerable to secondary injury mechanisms and cognitive deficits for an extended period following the initial TBI occurrence (42–46). Thus, we evaluated the microstructure of WM components and integrity of myelinated axons using immunostaining for MBP, LFB myelin staining, and electron microscopy in 6 months after rlmTBI. The progression of axonal degeneration was supported by the myelinated axonopathy (Fig. 2H, I, J, and L) and ultrastructural pathologies of axon and myelin degeneration (Fig. 2J and M) in the neocortex (and corpus callosum) of rlmTBI-induced p17KO males. Ultrastructurally, there were noticeable signs of demyelinated axons and disorganized myelin attachment to axons at paranodes within subcortical regions of the rlmTBI-induced p17KO males (Fig. 2J and M). In detail, most axonal mitochondria in rlmTBI-induced p17KO mice have compromised and disarranged membranes. We further observed that the ratio of AT8/Tau5 increased significantly in the neocortex of rlmTBI-induced p17KO males at 6 months postinjury (Figs. 2N and S5F–H), suggesting long-term effects of novel rlmTBI on the development of tau pathology in the subcortical regions. Behavioral findings were supported based on a histopathological assessment, and neither rlmTBI-induced p17KO nor WT female mice had signs of axonal pathology and mitochondrial changes (Fig. S7B, C, and E) or tau pathology (data not shown) in the neocortex at 6 months postinjury. Thus, the ablation of p17 in mice significantly contributes to mitochondrial dysfunction and axonal degeneration in the susceptibility to, and recovery from, long-term secondary neurological deficits associated with rlmTBI in a sex-dependent manner.

### Impaired p17/C18-Cer–associated mitophagy leads to the accumulation of dysfunctional mitochondria, which contributes to the susceptibility of secondary axonal degeneration following rlmTBI

**Fig. 3.**
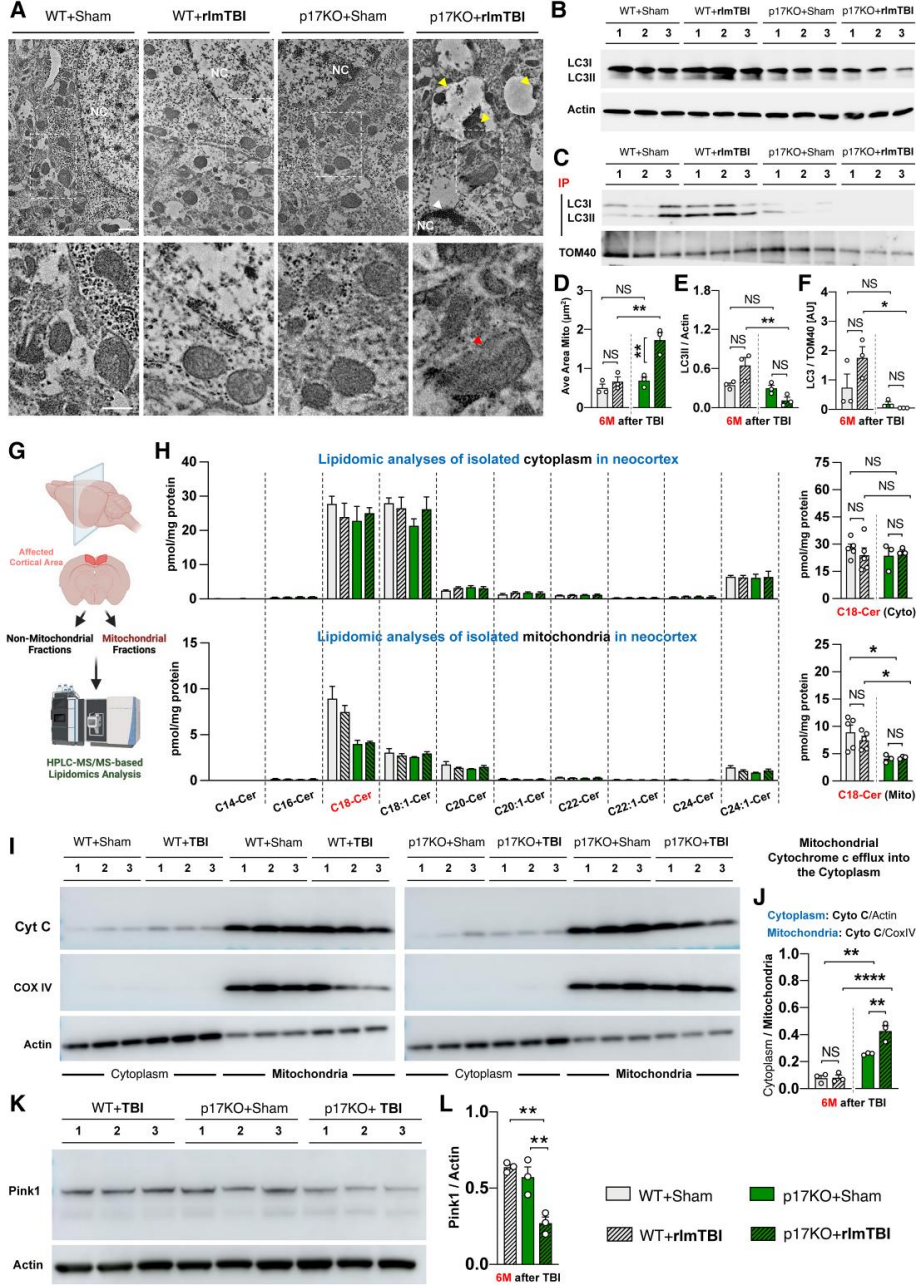
The accumulation of dysfunctional mitochondria due to impaired p17/C18-Cer–associated mitophagy contributes to secondary axonal degeneration after rlmTBI. A new cohort of 2-month-old male p17KO and C57BL/6J WT mice underwent rlmTBIs or sham injuries, followed by the analysis of mitochondrial function, structure, and intracellular organization at 6-month post-rlmTBIs. A) Ultrastructural pathologies of axonal mitochondria of the cortical neurons and (D) quantification (average area of the mitochondria) were determined. The red arrow head indicates tubular and large mitochondria that are likely to represent fusion at early stages of myelinated axonal mitochondria; the yellow arrow heads indicate autophagic vacuoles and engulfed mitochondria; the white arrow head indicates darkened nucleoplasm and a crenated nucleolemma of the oligodendrocyte. The below images exhibited the higher magnification of each corresponding dashed boxed area in the upper panels. Scale bars, 1 μm. B and E) The lipidated LC3 (LC3-II) was analyzed with immunoblotting in the neocortex. C and F) Using co-IP analysis, the association between LC3/TOM40 was analyzed to measure mitophagy. G) A schematic representation of the microdissection procedure used to isolate the affected cortical region. H) HPLC-MS/MS–based lipidomics analyses of bioactive ceramide profiles in mitochondrial and nonmitochondrial fractions isolated from pathologically relevant cortical regions. I and J) Mitochondrial and cytosolic Cytochrome c protein levels were analyzed with immunoblotting in the neocortex with anti-Cytochrome c antibody. Whole-cell lysates were analyzed for (K and L) PINK1 expression by immunoblotting in the neocortex. Loading standards were actin for homogenate and Cox IV for mitochondria (*n* = 3–5). Data are expressed as mean ± SEM (two-way ANOVA with Bonferroni's correction and one-way ANOVA with Dunnett's correction). NS, not significant. *P < 0.05, **P < 0.01, and ****P < 0.0001.

Ceramide, especially C18-Cer, a bioactive sphingolipid produced in response to cell stress and injury, and its synthesizing enzyme (CerS1) act as precursors to selective stress-mediated mitochondrial autophagy (20–23). To understand whether C18-mediated mitophagy is the vital mechanism governing the maintenance of axonal homeostasis contributing to the development and progression of secondary injury in rlmTBI-induced p17KO male mice, we screened for changes in the mitochondrial physiology/structure in cortical regions of the brain, including mitochondrial damage/dysfunction in neuronal axons and alteration in mitochondrial ceramides and mitophagy in a new cohort of mice. The ultrastructure of the cortical neuronal mitochondria in rlmTBI-induced p17KO male mice developed tubular and large mitochondria (size) that are likely to represent fusion in its early stages (Fig. 3A and D). Many mitochondria lost membrane integrity (Fig. 3A; red arrowheads). Autophagic vacuoles and engulfed mitochondria and other organelles appeared (Fig. 3A; yellow arrowheads). We noted a substantial number of neurons from p17KO-rlmTBI male mice undergoing dark degeneration, which was accompanied by increased numbers of autophagic vacuoles. Although rlmTBI-induced WT nor WT or p17KO sham male mice exhibited a pale nucleoplasm bound by a nucleolemma with a single or no indentation and a dense, centrally situated nucleolus, rlmTBI-induced male p17KO mice showed darkened nucleoplasm and a crenated nucleolemma of the oligodendrocyte (Fig. 3A; white arrowheads).

Lipidated LC3-II binds to accumulated mitochondrial ceramide to recruit the autophagosome for mitochondrial degradation (20, 23). The LC3 lipidation, known as LC3II, was significantly decreased in the neocortex collected from both sham- and rlmTBI-induced p17KO male mice, but not in WT controls, at 6 months post-rlmTBI (Figs. 3B and E and S8A and B). Co-immunoprecipitation (co-IP) analysis also verified that LC3/TOM40 (mitochondrial membrane protein) associations were significantly decreased in sham- and rlmTBI-induced p17KO male mice, compared with WT male counterparts at 6 months postrlmTBI (Figs. 3C and F and S8C). Notably, HPLC-MS/MS–based lipidomics analyses revealed that prevention of mitophagy in sham-induced and rlmTBI-induced p17KO male mice was consistent with reduced mitochondrial C18-Cer levels in the pathologically relevant affected cortical regions, compared with WT male controls (Fig. 3G and H). Release of cytochrome c from the mitochondria in response to cell stress or mitochondrial damage triggers neurodegeneration (47). Pink1 (PTEN-induced kinase 1) is a mitochondrially targeted serine/threonine kinase, and the down-regulation of Pink1 is intimately linked to mitochondrial dysfunction (48), which facilitates the opening of mitochondrial membrane permeability and releasing cytochrome c from the mitochondria (49–51). Notably, the neuroprotective activity of Pink1 could also be explained by the mitophagic removal of damaged mitochondria before they trigger neuronal death (52). In our present study, the ratio of cytosolic cytochrome c to mitochondrial cytochrome c levels was significantly increased in the neocortex collected from rlmTBI-induced p17KO male mice, but not from rlmTBI-induced WT nor WT or p17KO sham male mice, at 6 months post-TBI (Figs. 3I and J and S8A–F), which associates with reduced Pink1 expression (Figs. 3K and L and S9D and E). Loading standards were as follows: actin for cytoplasm and Cox IV for mitochondria. Consistent with the behavioral and pathological outcomes, rlmTBI-induced p17KO female mice did not show any significant loss in mitochondrial function and abnormal mitochondrial morphology compared with male counterparts in a time-dependent manner (Figs. S7B–H and S10).

Collectively, these data demonstrate the importance of the p17/C18-Cer axis for mediating ceramide-dependent mitophagy and for maintaining mitochondrial metabolism/homeostasis during secondary disease development after rlmTBI, which were altered in the subcortical region of p17KO mice.

### C18-Cer analog, LCL768, reinstitutes mitophagy and alleviates secondary disease development in p17KO mice following rlmTBI

Developing targeted pharmacologic interventions for TBI is a high priority, but finding the appropriate targets has proven elusive. Damaged mitochondria can accumulate due to impaired mitophagy, which is a central pathophysiological component in the secondary injury cascade of TBI. Mitophagy has gained attention as an essential mechanistic target for restoring steady-state levels of mitochondrial quality control and for removing damaged mitochondria. Mechanistically, outer mitochondrial membrane localization of C18-Cer plays a critical role in the recruitment of autophagosomes to damaged mitochondria via associating with LC3 involving its F52 residue (20, 23, 53). We have developed a novel mitochondrial-targeted pyridinium-ceramide (Pyr-Cer) analog drug, LCL768, that can accumulate specifically in damaged mitochondria to restore mitophagy in injured cells/tissues. Notably, LCL768 contains a positive charge at a delocalized pi-electron system, which then results in preferential localization of the drug into highly negatively charged mitochondria in injured cells/tissues due to the Warburg effect (54). Our very recent study has demonstrated that LCL768 selectively induced the CerS1 trafficking from mitochondria-associated membranes to the outer mitochondrial membrane, which does not require the p17 transporter but involves tethering ER and mitochondria in the brain, both in vitro and in vivo (25).

**Fig. 4.**
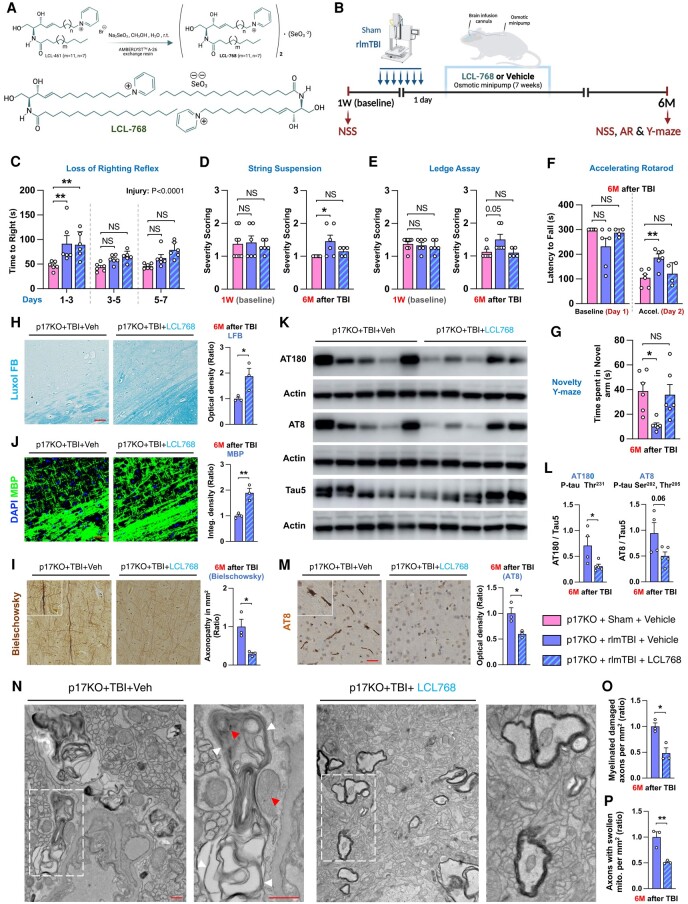
C18-Cer analog, LCL768, restores mitophagy and reduces secondary disease development in p17KO mice after rlmTBI. A) The chemical structure of LCL768 contains two C18-Pyr-Cer moieties conjugated with selenium. B) Experimental setup. Two-month-old male p17KO mice underwent rlmTBIs or sham injuries, and 1 day after the last injury, they were chronically treated with LCL768 (0.1 mg/kg) or vehicle (PBS) through intracranially (inserted directly into the overlaying cortex) implanted osmotic minipumps (Alzet) for 7 weeks. Treatment began 1 day after their last injury and continued for 7 consecutive weeks with 3 months of washout. C) The loss of the righting reflex was evaluated based on the latency of self-righting immediately after the injuries. Their neurological competency (NSS and AR) was longitudinally assessed by (D) Ledge assay, (E) string suspension, and (F) accelerated rotarod tests in mice before injury and 6 months after the last injury. (G) Working memory performance was assessed with the novelty Y-maze in mice 6 months after the last injury. The microstructural organization of myelinated axons and axonal integrity were assessed by immunostaining for (H) LFB, (J) MBP myelin, and (I) Bielschowsky silver staining. Bar graph showing the quantification of optical density of LFB staining and integrated fluorescence density of MBP immunostaining in the neocortex of mice in each group (*n* = 3). Scale bars, 50 μm. K and L) Western blot analysis of cortical tau pathology was analyzed for AT8, AT180, and Tau5 (total tau) at 6 months after rlmTBIs (*n* = 4–5). M) AT8-positive pathological tau was also assessed by immunohistology (*n* = 3). N) Electron microscopy (red arrows indicate degenerated axonal mitochondria; white arrows indicate disorganized myelin attachment to axons at paranodes; Scale bar, 500 nm) in the neocortex 6 months after the last injury. Inset images are high magnifications of representative areas. Bar graph showing the quantification of the relative (O) myelinated damaged axons and (P) axons with swollen mitochondria at EM imagining in the neocortex of mice in each group (*n* = 3). Data are expressed as mean ± SEM (one-way ANOVA with Dunnett's correction). NS, not significant. *P < 0.05 and **P < 0.01.

In this study, we set up a rescue experiment to investigate whether pharmacological intervention utilizing a ceramide analog and a mitophagy inducer, LCL768, which circumvent the p17/CerS1 axis, will restore mitophagic quality control and attenuate the development of secondary deleterious cascades following brain injuries in rlmTBI-induced p17KO mice. The p17KO-rlmTBI mice were randomly treated with either LCL768 (0.1 mg/kg; Fig. 4A) or vehicle (phosphate-buffered saline, PBS) using Alzet osmotic minipumps directly inserted into the overlaying cortex in a double-blinded manner. The treatment started a day after the last injury and lasted for 7 weeks, followed by a 3-month washout period without any treatment (Fig. 4B), as described in the Materials and methods section. Long-term functional outcomes were assessed at 6 months after injury after which mice were examined for histopathological outcomes. The loss of the righting reflex was evaluated based on the latency of self-righting immediately after the injuries (Fig. 4C). Treatment with LCL768 prevented the development of neurological and cognitive deficits following rlmTBI as detected by string suspension (Fig. 4D), Ledge assay (Fig. 4E), accelerated rotarod (Fig. 4F), and novelty Y-maze (Fig. 4G). Moreover, treatment with LCL768 eliminated the induction of WM degeneration (Fig. 4H and J) and axonal pathology (Fig. 4I) and prevented tau pathology (Figs. 4K–M and S11) across the cortical regions of the brain. Notably, transmission electron microscopy revealed a significant increase in well-organized mitochondria and a decrease in demyelinated axons in subcortical regions of p17KO-rlmTBI mice treated with LCL768 compared with vehicle-treated controls (Fig. 4N–P). These results suggest that treatment with a mitophagy inducer LCL768 can prevent the development of secondary axonal degeneration by eliminating damaged mitochondria, which leads to improved functional outcomes after rlmTBI.

### CTE patients have diminished access to p17/CerS1/C18-Cer–mitophagy axis, resulting in impaired mitochondrial stress response in subcortical regions of the brain

CTE is strongly associated with repeated head injuries, particularly concussions or subconcussive hits (1, 55–57). However, the specific sequence and interaction of mechanisms involved in CTE are still mostly unknown. Several factors may impact the development and advancement of the condition (58), e.g. genetic predisposition and individual susceptibility. Thus, research to uncover the pathogenesis of CTE and to find possible targets for intervention therapy is crucial to the field (26, 59–61).

**Fig. 5.**
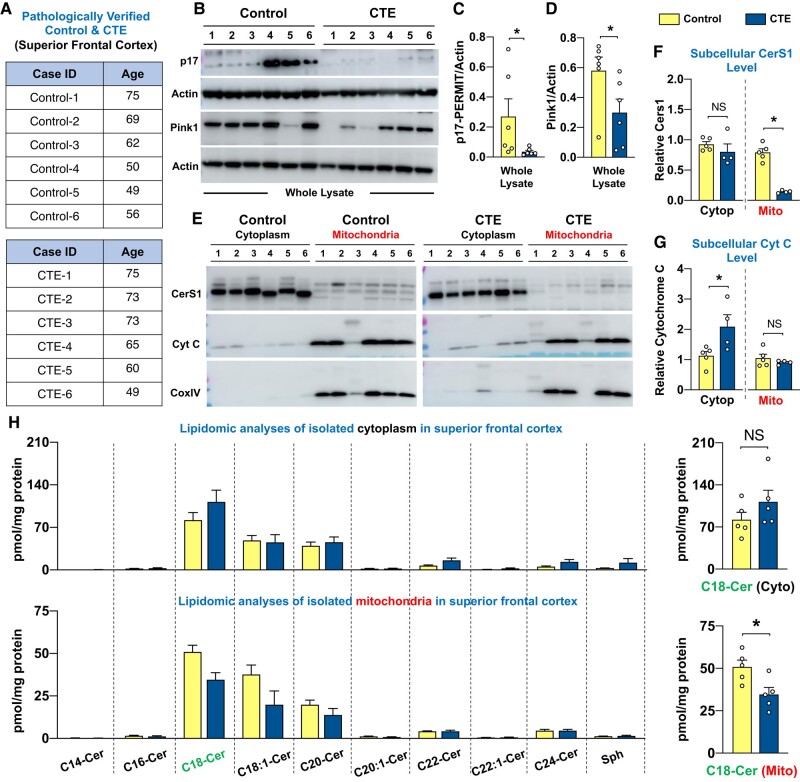
Significant alterations of the p17/CerS1–mitophagy axis in patients with CTE. A) The six neuropathologically verified male CTE brain specimens of the superior frontal cortex under the age of 75 and six age-matched healthy controls were subjected to immunoblot and lipidomic analysis of subcellular fraction. Whole-cell lysates were analyzed for (B and C) p17 and (B and D) PINK1 expressions by immunoblotting in the superior frontal cortex. Mitochondrial and cytosolic (E and F) CerS1 and (E and G) Cytochrome c protein levels were analyzed with immunoblotting in the neocortex with anti-Cytochrome c and CerS1 antibodies, respectively. H) Mass spectrophotometry–based lipidomics analyses of cytoplasmic and mitochondrial C^14^–C^26^-ceramide levels in the superior frontal cortex. Loading standards were actin for homogenate and Cox IV for mitochondria (*n* = 6). Data are expressed as mean ± SEM (two-way ANOVA with Bonferroni's correction). NS, not significant. *P < 0.05 and **P < 0.01.

To support the premise that the p17-mediated mitochondrial stress response and mitophagy are a clinically relevant, endogenous neuroprotective response following rlmTBI, we examined the six neuropathologically verified CTE cases with a history of repetitive TBI (<75 years of age) and compared with six age-matched and sex-matched healthy control samples (Fig. 5A) (1). Notably, our human data demonstrated that there was a significant loss of p17 expression (Figs. 5B and C and S12A and B), accompanied by a considerable reduction of mitochondrial CerS1 accumulation (loading standards were actin for homogenate and Cox IV for mitochondria; Fig. 5E and F) and mitochondrial C18-Cer (Fig. 5H) in CTE human brains compared with age-matched control subjects. Our results further revealed that compared with non-CTE health controls, the ratio of cytosolic cytochrome c to mitochondrial cytochrome c levels was significantly increased in the temporal cortex collected from CTE patients (Figs. 5E and G and S12C–K), which associates with reduced PINK1 expression (Figs. 5B and D and S12L and M).

Thus, these data suggest that impaired mitochondrial stress response and defective mitophagy in CTE patients with a history of repetitive TBI may be, in part, attributed to alterations in the p17/CerS1–mitophagy axis. These findings support the clinical relevance of this mechanism in neurodegeneration disorders associated with cognitive deficits, including CTE.

## Discussion

It is currently unclear whether the neuropathology observed in CTE is directly associated with a history of exposure to repetitive mild head injuries or whether it is linked to other confounding factors that cause resistance and resilience to neuropathology and clinical disease. To assist in elucidating those potential factors, the present study is the first to introduce a new experimental model, *rlmTBI* paradigm, to demonstrate how additional intrinsic/extrinsic confounding factors cause resistance and resilience to the neuropathology of CTE. We first demonstrated the long-term pathological and behavioral impacts of existing rmTBI and new rlmTBI in 2-month-old WT mice. In both rmTBI and rlmTBI mice, the average time taken for recovery of the righting reflex was significantly higher in comparison with the sham controls. However, during the first 3 days, the mice exposed to rmTBI took significantly longer to self-right than those exposed to rlmTBI. At 8 months postinjury, the existing rmTBI model induced significant neurological and cognitive deficits. These deficits were accompanied by progressive degeneration of WM and axonal tau pathology in good accordance with our previous reports (26, 27, 29, 59). In contrast, receiving rlmTBI at 2 months of age did not result in any long-term impairments. Since the aged brain is more prone to develop secondary diseases after rmTBI than the young brain, we further tested this new rlmTBI model in the brains of 2-month-old (young) and 12-month-old (old) WT mice. The brains of aged WT mice (12 months old at the time of injury) exhibited notable long-term degeneration of WM and tau pathology in the subcortical regions associated with cognitive impairments during the secondary injury phase following rlmTBI at 8 months postinjury. This phenomenon was not observed in younger WT mice brains (2 months old at the time of injury).

Increasing evidence supports that mitochondrial maintenance and mitophagy are key players in the pathogenesis of age-associated neurodegenerative disorders (19, 62, 63). However, the causal role of mitochondrial quality control and proper mitophagy in secondary disease development after rmTBI (13, 14) and its clinical relevance remain relatively unknown. Here, we demonstrate, for the first, that p17/C18-Cer–mediated mitophagy alteration significantly contributes to mitochondrial dysfunction and axonal degeneration in the susceptibility to, and recovery from, long-term secondary cognitive deficits associated with novel rlmTBI. We further showed that a novel mitochondrial targeted Pyr-Cer analog and a mitophagy inducer drug, LCL768, can prevent the development of secondary axonal degeneration by maintaining a healthy pool of axonal mitochondria, leading to improved functional outcomes following rlmTBI.

**Fig. 6.**
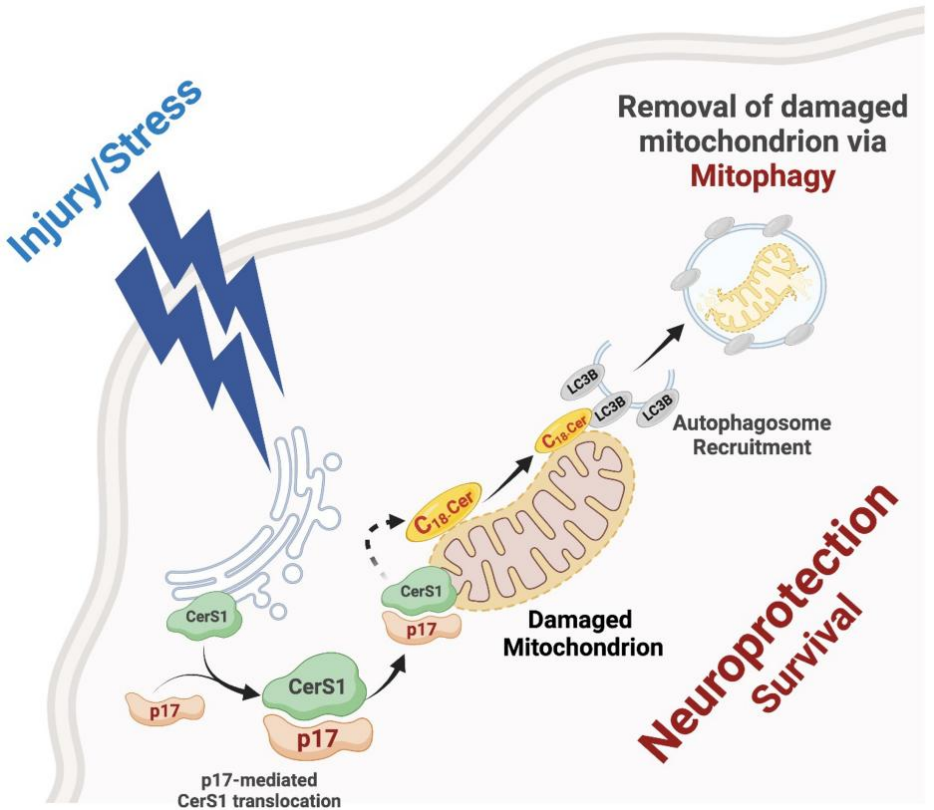
p17/C18-Cer–mediated mitophagy is an endogenous neuroprotective mitochondrial stress response at the time of brain injury and beyond. Subcellular localization of CerS1 by the novel p17 transporter in damaged mitochondria vs. ER to induce C18-Cer generation, and the subsequent injury/stress requires LC3 activation and mitophagy in various metabolically active tissues, including the brain.

Notably, our results also suggested that the impaired mitochondrial stress response and defective mitophagy in CTE patients (under the age of 75) with a history of repetitive TBI may be, in part, attributed to alterations of the p17/CerS1/C18-Cer–mitophagy axis. Together, our current results support the idea that p17 drives mitochondrial CerS1 trafficking to induce C18-Cer–mediated mitophagy, thus ensuring mitochondrial quality control by maintaining a healthy pool of axonal mitochondria in the brain (Fig. 6), which, in turn, may contribute to a susceptibility to developing progressive neurodegenerative diseases post-rmTBI, including CTE.

Mitophagy is responsible for the ultimate recycling of dangerous, dysfunctional mitochondrial components. This mechanism may have potential benefits, especially in the case of injured neuronal axons. By reducing the contribution of mitochondria to harmful redox cycles, it could potentially contribute to the recovery and healing of nerve cells (64, 65). Yet, due to the complexity of mitophagy mechanisms, mitochondrial autophagy is both detrimental and beneficial (66–70). Thus, it is possible that the beneficial vs. detrimental effects of mitophagy after TBI depend on injury type and timing after injury. Therefore, further research is needed to determine whether mitophagy can specifically improve mitochondrial quality in axons and neurons and therefore ultimately play a neuroprotective role against secondary disease development after TBI.

Moreover, an increasing number of studies in rodents and humans have demonstrated significant sex differences in response to mild TBIs, in which females show reduced susceptibility to secondary injuries and have overall better outcomes after primary injury compared with males (71–74). Hence, accumulating evidence suggests that the propensity of the mitochondrial stress response and mitophagy may vary between males and females, particularly under cellular stress conditions such as with TBI (75–78). However, it remains elusive how sex differences in the mitophagic response contribute to the development and progression of disease in rmTBI. The present study demonstrated that ablation of the mitochondrial p17/C18-Cer–mediated mitophagy response worsened the secondary neurological insults after rlmTBI in males, but not in females, indicating that the p17/C18-Cer–mediated mitochondrial stress response is crucial in the sex-specific secondary disease mechanisms following repetitive concussive TBIs. A combination of biological factors such as sex differences contribute to the secondary sequelae that determine the long-term cognitive deficits after injury. The biological underpinnings of sexual dimorphism have traditionally centered on the sex chromosomes and the production of sex hormones that interact with cellular receptors (76, 79). Notably, recent reports indicate mitochondria are targets of 17beta-estradiol, a critical female hormone (80–82), which might act directly on mitochondrial integrity as a neuroprotective agent following TBI.

On the other hand, recent reports have demonstrated that sexual dimorphism exists independent of hormonal differences, particularly in the changes to mitochondrial structure and function and their impact on the cellular stress response in neurodegenerative conditions (79). For example, early animal studies showed that the estrous cycle stage at the time of TBI did not affect brain contusion volumes or functional outcomes in female rats (83, 84). In addition, newborn male piglets had significantly higher secondary cerebral damage, including impairment of cerebral autoregulation, after TBI compared with newborn females, despite their sexually immature state (85). Human studies also showed that the estrous cycle stage and the use of oral contraceptives did not affect postconcussion syndrome, including balance deficits and postural instability (86–88). Those preclinical or clinical studies, along with the failure of two major phase III trials of progesterone treatment for TBI (89, 90), indicated that factors beyond sex hormones are likely to be important contributors to the sexual dimorphism in secondary damage after TBI.

There are several limitations to our present study. First, the study focused on the involvement of p17/C18-Cer–mediated mitophagy in the mitophageal elimination of damaged mitochondria; this study did not explore the possible contribution of other mitophagy pathways cardiolipin (64, 91), PINK1/Parkin (92–94), and BNIP3L/NIX (95, 96) to TBI-induced mitophagy in the brain. Second, we used a new experimental model of repetitive concussive injury, whereas controlled cortical impact (CCI) is another widely used and validated severe contusion injury model. In this study, we focus on the pathogenesis of CTE, a progressive degenerative disease affecting people who have suffered repeated concussive injuries, not single contusion injuries. Yet, exploring the effects of p17/C18-Cer–mediated mitophagy in the CCI model would be valuable. Last, although we highly appreciate obtaining human brain samples for our study, it was not feasible to obtain a significant number of neuropathologically verified CTE female brains. Indeed, it is only very recently that the Australian Sports Brain Bank identified the first case of CTE in females worldwide during an autopsy (97).

In summary, regardless of the limitations, our new experimental model of CTE (repetitive less-than-mild closed head injury [rlmTBI] paradigm) allowed us to develop insights into how the brain becomes more susceptible to developing secondary sequalae after a repetitive concussion, or mild TBI, and that p17/C18-Cer–mediated mitophagy alterations may play a significant role in the extent of the neuropathology of CTE.

## Materials and methods

### Animals

Mice were housed in group cages (*n* = 4 per cage) containing bedding covering the floor, overhead storage of food pellets, and a water bottle for the mice to feed and drink freely. The animal facility where the mice were housed was maintained at a constant temperature and humidity, and the animals were subject to a standard 12-h light–dark cycle. All animals used were male to control for changes to cognition occurring in different hormonal states in female mice. All experiments comply with the relevant guidelines and regulations regarding the care and use of animals for experimental procedures in accordance with the standards of the International Animal Care and Use Committee and the Medical University South Carolina (MUSC) Animal Care and Use Committee.

### Alzet pumps’ intracranial implantation and LCL768 treatment

Two-month-old rlmTBI-induced p17KO male mice were chronically treated with LCL768 (0.1 mg/kg) or vehicle (PBS) through intracranially (inserted directly into the overlaying cortex) implanted osmotic minipumps (Alzet, CA, USA) (98) for 7 weeks. The animals were chosen at random to participate in the treatment groups. Treatment began 1 day after their last injury and continued for 7 consecutive weeks. After the treatment period was completed, there was a 3-month washout period where no treatment was administered. To begin the treatment, the pump was assembled and loaded with LCL768 (0.1 mg/kg; 0.15 µL/h for 42 days). The pump was then equilibrated for 48 h at 37 °C before intracranial implantation, which was done according to the manufacturer's instructions.

### Human brain specimens

Fresh-frozen human brain tissue from the superior frontal cortex of individuals with neuropathologically verified CTE was provided from the VA-BU-SLI Brain Bank of the Boston University Alzheimer's Disease Center CTE Program, including six patients with a history of exposure to TBI and six age-matched healthy controls (Table S1) (1). Next of kin provided written consent for participation and brain donation. Institutional review board approval for brain donation was obtained through the Boston University Alzheimer's Disease Center, CTE Program, and the Bedford VA Hospital. Institutional review board approval for neuropathological evaluation was obtained through the Boston University School of Medicine (1). Our studies on human samples have been approved by our Institutional Review Boards at Boston University and the Medical University of South Carolina.

### rlmTBI and rmTBI models

Male C57BL/6J mice (2-month-old and 12-month-old) obtained from the Jackson Laboratories (Bar Harbor, ME) and male p17KO mice (2-month-old) were randomized to undergo injury or sham-injury. The mice were anesthetized for 5 min using 4% isoflurane in a 70:30 mixture of air:oxygen. Anesthetized mice were placed on a delicate task wiper (Kimwipe, Kimberly-Clark, Irving, TX, USA) and positioned such that the head was placed directly under a hollow guide tube. The mouse's tail was grasped. A 54-g metal (tungsten) bolt was used to deliver an impact to the dorsal aspect of the skull, resulting in a rotational acceleration of the head through the Kimwipe. Mice underwent seven repetitive less-than-mild injuries (rlmTBI, 24-inch height; W/5 mm flat) OR repetitive mild injuries (rmTBI, 36-inch height; W/5 mm flat) in 9 days. Sham-injured mice underwent anesthesia but not concussive injury. All mice were recovered in room air. Anesthesia exposure for each mouse was strictly controlled for 5 min. Briefly, anesthetized mice were exposed to a less-than-mild hit or sham hit, removed from the apparatus, monitored until the recovery of gross locomotor function, and then transferred to their home cage. Maximum burst pressure was compatible with 100% survival, and no gross acute motor abnormalities were ascertained empirically. All these and the following animal experiments were approved by the Medical University of South Carolina and IACUC and complied with the NIH Guide for the Care and Use of Laboratory Animals.

### Behavioral tests

All the tests were conducted from 10 AM to 3 PM in the lights-on cycle at MUSC Animal Research Facility or Veteran Affairs Small Animal Behavioral and Physiological Assessment Core by experimenters blinded to group designation. Mice were habituated to the procedure room 30 min before each test.

### Ledge assay

In the ledge test, mice were placed on the elevated cage's ledge at a height of 35 and 0.8 cm wide and monitored their movement as previously established (26, 99). Each mouse was tested three times (each test takes 20 s) and scored from 0 to 3 depending on the severity of deficits in a double-blind manner. Scoring is as follows: if the mouse walked along the ledge, without foot faults (i.e. loosing footing) and back into the cage delicately, the score is 0; if the mouse demonstrated any foot fault while walking on the ledge, the score is 1; if the mouse did not effectively walk on or dismounted the ledge immediately, the score is 2; and if the mouse fell off the ledge or avoided walking, the score is 3.

### String suspension assay

The mouse was permitted to grasp a string only by its forepaws suspended 35 cm above the surface and was then released as previously established (26, 99, 100). Each mouse was tested three times (each test takes 20 s) and scored from 0 to 3 depending on the severity of deficits in a double-blinded manner. If the mouse was unable to remain on a string, the score was 3; if it hung by both forepaws and attempted to climb onto the string, the score was 2; if both forepaws and one or both hindpaws were around the string, the score was 1; and if four paws and tail were around the string, with lateral movement, the score was 0.

### Accelerating rotarod test

The mice were placed in the rotating cylinder three times per day for 2 consecutive days in total. Each trial lasted a maximum of 5 min, during which time the rotating rod accelerated from 4 to 40 rpm. over the first 1 min of the trial and then remained at the maximum speed for the remaining 2 min. Animals were rested for at least 10 min between trials to avoid fatigue and exhaustion.

### Bright light, open-field test

Mice were placed in the center of the open-field apparatuses of brightly lit (200–300 lx) chambers (44 × 44 × 30 cm). Movements of the animals were tracked by a computer-assisted video-tracking system (Noldus Ethovision XT) for 10 min. Horizontal motor (distance traveled) and central activity (distance traveled in the center/total distance traveled) were evaluated.

### Novelty preference test (Y-maze)

Mice were longitudinally tested in novelty Y-maze, which consisted of three closed arms in the Y-shape (50 cm × 11 cm × 10 cm) made of white Plexiglas. This test includes two sessions. During a training session, mice were pseudo-randomly assigned the two arms (the “start arm” and the “left or right arm”), allowing for a 3-min exploration of only these two arms of the maze. After a 1-min delay, a test session was started. During the test session, the mice were allowed to explore freely all three arms of the maze for 3 min. The test session takes advantage of the innate tendency of mice to explore novel unexplored areas (e.g. the previously blocked arm). A computer-assisted video-tracking system (Noldus Ethovision XT) recorded the time spent by each animal in novel unexplored areas of each animal. Mice were first tested 1-month after the last injury and then were retested 6 months later in the same maze but in a different environment. Mice with intact short-term memory prefer to explore a novel arm over the familiar arms, whereas mice with impaired spatial memory enter all arms randomly. Thus, the test session represents a classic test for spatial working memory as previously described (27, 98, 101).

### Barnes maze test

Barnes maze is a memory task that requires mice to use spatial cues around an elevated platform to locate a hidden goal box as described previously (102). Cages were brought into the behavior suite 30 min prior to starting. The animals were exposed to bright light (clamp lamp positioned above the maze, 100 W) throughout testing. To help increase the motivation to enter the escape box, bedding from each cage was placed inside. Each mouse was placed in the middle of the maze at the start of each trial and allowed to explore for 3 min. Each trial ended when the mouse entered the escape box or after 3 min had elapsed. Immediately after the mouse entered the box, it was allowed to stay there for about 30 s. If the mouse did not reach the goal within 3 min, then the experimenter gently guided the mouse to the escape box and left the mouse inside for about 30 s. Once the mouse was placed back in its home cage, the maze and escape box were cleaned with 70% ethanol followed by testing of the next mouse. This was repeated until each animal had received three trials per day over 5 consecutive days. During these training days, the latency to the goal, distance traveled, and time in the goal perimeter were recorded and traced with a computer-assisted video tracking system (Noldus Ethovision XT).

### Ultra-structural analysis using TEM

Mice were perfused with a fixative solution, a mixture of 15% picric acid (13% saturated solution; Sigma, St. Louis, MO, USA), 4% paraformaldehyde (Electron Microscopy Sciences, Hatfield, PA, USA), and 0.1% glutaraldehyde (EM grade 50% solution; Electron Microscopy Sciences) dissolved in a general tubulin buffer (PEM) (0.1 M PIPES, pH 7.2, 1 mM EGTA, and 1 mM MgCl_2_). Perfused brains were removed, sliced, and kept in the same fixative for further 4 h at 4 °C. The samples were processed for electron microscopic observation as described. Specimens were examined with a JEM-1010 transmission electron microscope (JEOL) (103).

### HPLC-MS/MS analysis of sphingolipids

We conducted an HPLC-MS/MS–based lipidomics analysis of bioactive ceramide profiles in mitochondrial and nonmitochondrial fractions isolated from pathologically relevant cortical regions. We selected the affected cortical area, which was ∼5 cm^3^ in size and contained roughly 2.5 × 10^5^ neurons, based on the presence of robust axonal pathology. We have dissected the same cortical region in the control groups according to the Allen Brain Reference Atlas. Lipid extractions and analyses were performed by Lipidomics Shared Resource, Analytical Unit, MUSC. Briefly, cells were lysed with radioimmunoprecipitation assay (RIPA) buffer. Further preparation of samples and advanced studies of endogenous bioactive sphingolipids were performed on ThermoFisher TSQ Quantum liquid chromatography/triple-stage quadrupole mass spectrometer system, operating in multiple reactions monitoring positive ionization mode, as previously described. Lipid levels were normalized to the protein level present in samples (pmol/mg protein) (24).

### Antibodies

The antibodies used for western blotting and immunohistology in this study were as follows: COXIV (ab160561, Abcam), Tom40 (sc-365467, Santa Cruz Technology), PINK1 (D8G3, 6946, Cell Signaling Technology), LC3B (2775S, Cell Signaling Technology), p17 (Ribosomal Protein L29 [P-14], sc-103166 Santa Cruz Biotechnology), P-tau^Ser^202^/Thr205^ (AT8—MN1020 Thermofisher), P-tau^Thr231^ (AT180—MN1040 Thermofisher), anti-Tau [TAU-5] (ab80579, Abcam), MBP (ab218011, Abcam), anti-β-actin-peroxidase antibody (A3854, Millipore Sigma), Cytochrome c (ab65311, abcam), and CerS1 (MBS2523738, MyBiosource).

### Western blotting

Briefly, equivalent amounts of cell lysates (9 μg protein/lane) were loaded onto 4–12% Bis-Tris gel; proteins were separated and transferred to nitrocellulose membranes. The membranes were blocked with 5% nonfat milk followed by incubation overnight at 4 °C with target antibodies at 1:2,000 dilution in 5% BSA or anti-β-actin at 1:10.000 dilution in 5% bovine serum albumin (BSA). After washing, membranes were incubated for 1 h at room temperature with appropriate secondary antibodies (horseradish peroxidase [HRP]–conjugated; dilution 1:5,000). Prestained molecular weight markers were run in parallel to identify the molecular weight of proteins of interest. For chemiluminescent detection, the membranes were treated with an enhanced chemiluminescent reagent, and the signals were monitored on Amersham imager 680 (GE Healthcare Bio-Sciences Corp., Marlborough, MA, USA). Relative band intensity was determined by densitometry using Image-J and normalized with β-actin protein.

### Immunoprecipitation

Cellular lysates in RIPA buffer containing a protease inhibitor cocktail (Sigma-Aldrich) were normalized by the total protein level and analyzed by SDS–PAGE and immunoblotting with corresponding antibodies. For immunoprecipitation, precleared cytosolic fractions were incubated overnight with 2 μg of corresponding antibody (Tom40 [D-2] [SC365467, Santa Cruz]) at 4 °C, followed by 1-h incubation with Protein A/G Agarose (Santa Cruz Biotechnology; 50 μL of a 50% slurry). Resin was washed three to five times, and pulled-down proteins were analyzed by SDS-PAGE and western blotting with corresponding antibodies (LC3B [2775S, Cell Signaling Technology]).

### Immunostaining

Immunostaining analysis was carried out as follows: upon animal sacrifice, the whole brain was collected and placed for 24 h at room temperature (RT) in 10% formaldehyde solution, followed by incubation in 70% ethanol at 4 °C overnight. The primary antibody used was anti-MBP (ab218011, Abcam) at 10 μg/mL concentration. After deparaffinization and rehydration, slides were briefly boiled in Tris-EDTA buffer, for antigen enhancement. The sections were incubated with primary antibody solution overnight at 4 °C. For double immunofluorescence staining, the sections were incubated with Alexa Fluor 488 or 594 conjugated isotype-specific secondary antibodies (Invitrogen A11008 and A11032) for 1 h at room temperature. Goat serum (50062Z, Invitrogen) as a blocking agent was used for each reaction. The sections were washed four times with PBS after each step. Labeled sections were visualized with a Zeiss confocal microscope or with the Keyence X800 and analyzed with Image J.

### Bielschowsky silver staining

Sections (10 μm thick) of paraformaldehyde-fixed and paraffin-embedded tissues were deparaffinized and then received silver solutions 1, 2, and 3 subsequently according to the following protocol (104), followed by 5% natrium thiosulfate in water and dehydration through a graded series of EtOH (70%, 90%, 100%), for 5 min each, and then clear slides in two changes of xylene solutions. Sections were then covered with mounting media and cover slipped. The optical density was measured using Fiji/ImageJ Coloc 2.

### LFB staining

After hydration with 95% alcohol for 5 min, slides were incubated in LFB solution (FD NeuroTechnologies) overnight at 60 °C followed by lithium carbonate solution washing for 5 min at room temperature and two 10-min washes in 70% ethanol. Sections were then rinsed with dH_2_O and covered with mounting media and cover slipped. The optical density was measured using Fiji/ImageJ Coloc 2.

### Mitochondria isolation

The mitochondrial isolation kit (ab65320) protocol was followed. Briefly, frozen cortex tissues wee thawed on ice and washed with frozen cold PBS once. Tissues were homogenized on ice with a sonicator. Approximately, 30–50 passes were performed with the sonicator. To check the efficiency, 2–3 μL of the homogenized suspension was pipetted onto a coverslip and observed under a microscope. A shiny ring around the cells indicates that the cells are still intact. If 70–80% of the cells did not have the shiny ring, then we proceeded to the next step. Otherwise, 10–20 additional passes were performed using the sonicator. Homogenates were transferred to a 1.5-mL microcentrifuge tube and centrifuged at 3000 rpm in a microcentrifuge for 10 min at +4 °C. The supernatants were carefully collected, and the pellets were discarded. Next, the supernatants were transferred to a fresh 1.5-mL tube and centrifuged at 10,000 *×g* (∼13,000 rpm) in a microcentrifuge for 30 min at +4 °C; the supernatant was collected and saved as “cytosolic fraction.” Then, the pellets were resuspended with 100 μL of the Mitochondrial Extraction Buffer Mix containing dithiothreitol (DTT) and protease inhibitors, vortexed for 10 s, and saved as “mitochondrial fraction” (stored at −80 °C).

### Cytochrome c release assay

Cytochrome c Release Assay Kit (ab65311, Abcam) protocol was followed. Briefly, after the mitochondria isolation, cytosolic and mitochondrial fractions of protein lysates of the same samples were run on the 4–12% Bis-Tris gel; proteins were separated and transferred to nitrocellulose membranes. The membranes were blocked with 5% BSA followed by incubation overnight at 4 °C with anti-Cytochrome c mouse antibody (1 μg/mL) prepared in 5% BSA. In order to further confirm the mitochondrial isolation efficiency, membranes were incubated with COXIV Mouse Antibody (ab14744, Abcam) at 1:1,000 dilution prepared in 5% BSA overnight at 4 °C. After washing, membranes were incubated for 1 h at room temperature with appropriate secondary antibodies (HRP-conjugated; dilution 1:5,000). Prestained molecular weight markers were run in parallel to identify the molecular weight of proteins of interest. For chemiluminescent detection, the membranes were treated with an enhanced chemiluminescent reagent, and the signals were monitored on Amersham imager 680 (GE Healthcare Bio-Sciences Corp.). Relative band intensity was determined by densitometry using Image-J and normalized with β-actin protein.

## Supplementary Material

pgae018_Supplementary_Data

## Data Availability

All data are included in the manuscript.
