# Supplementary material for: p17/C18-ceramide–mediated mitophagy is an endogenous neuroprotective response in preclinical and clinical brain injury
Source: PNAS Nexus. 2024 Feb 7;3(2):pgae018. doi: 10.1093/pnasnexus/pgae018 (PMC10847724; doi:10.1093/pnasnexus/pgae018)
Supplement: pgae018_Supplementary_Data [file pgae018_supplementary_data.pdf]

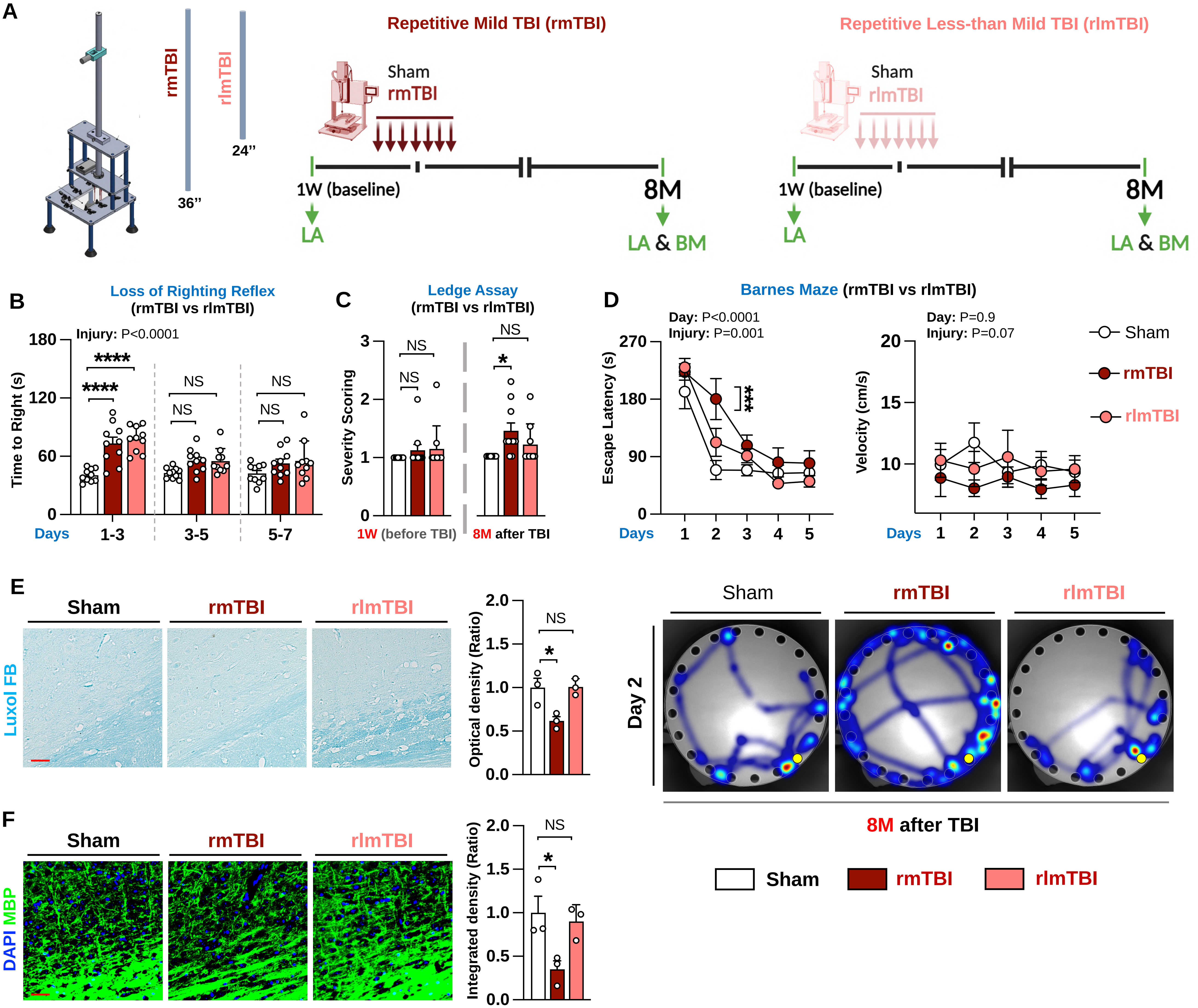

Supplementary Figure S1. (A) Experimental setup. Two-month-old male C57BL/6J WT mice underwent rmTBI, rlmTBI or sham injuries, and **(B)** the latency of their righting reflex was recorded, followed by functional and pathological examination for 8 months. The long-term effect of the injuries on neurological deficits were longitudinally assessed by **(C)** the Ledge Assay (LA) in mice before rlmTBI, and 8 months after the last injury. **(D)** Cognitive performance assessed by Barnes maze (BM) 8 months after the last injury, escape latency (s) and velocity (cm/s) across the 5 days of acquisition phase (above) and movement heat map on day 2 (the occupancy rate is graded by a color map ranging from cold to warm colors). The long-term neuropathological consequences of rmTBI and rlmTBI on the white-matter degeneration, as shown with LFB staining **(E)** and by immunofluorescence (IF) for MBP **(F)** to myelinated axonopathy in neocortex and corpus callosum. Scale bar, 50 µm. Data expressed as mean±SEM (two-way ANOVA with Bonferroni's correction and one-way ANOVA with Dunnett's correction). NS: not significant. *P < 0.05, **P < 0.01, ***P < 0.001, and ****P < 0.0001.

Supp. Figure 1

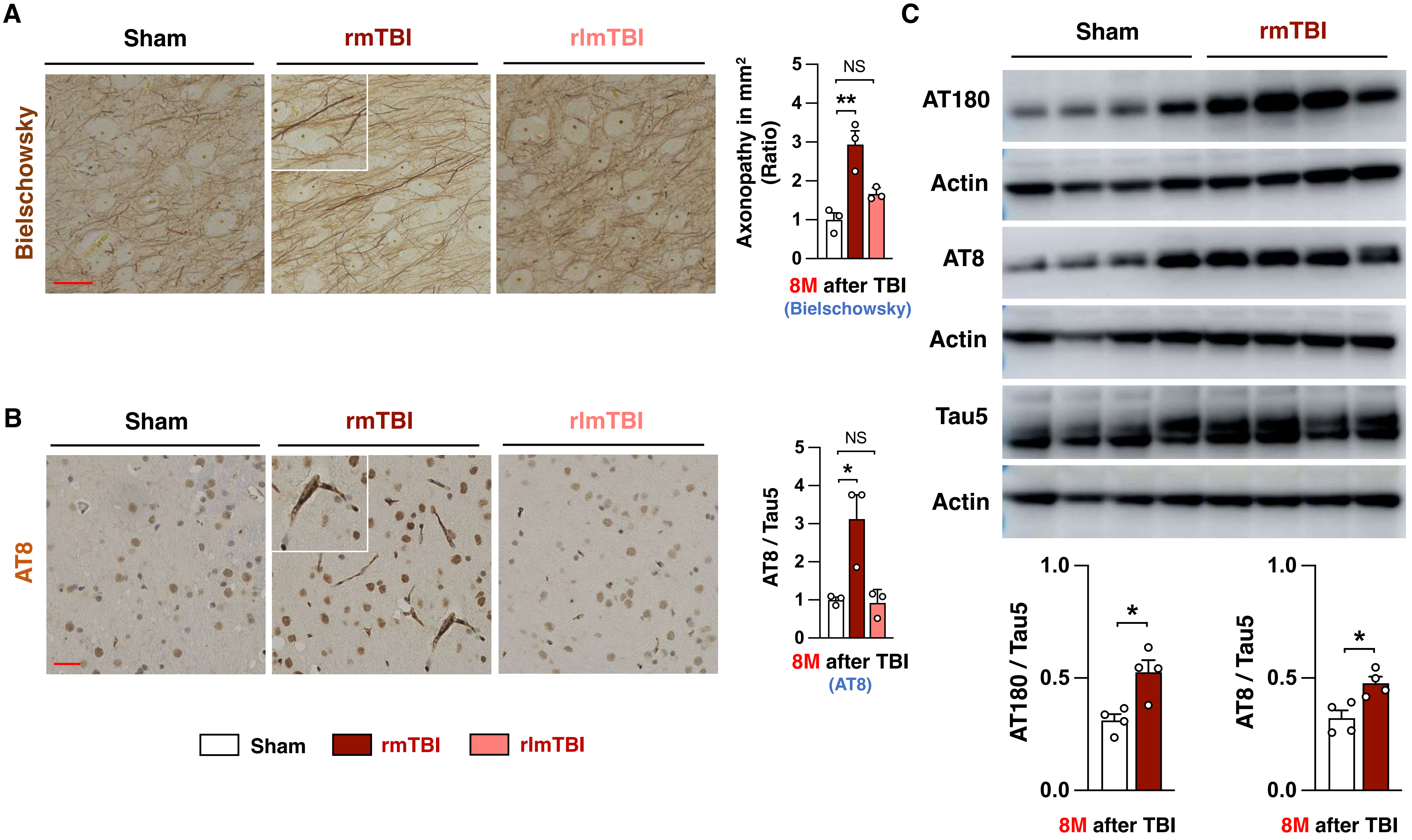

Supplementary Figure S2. The long-term neuropathological consequences of rmTBI and rlmTBI on the axonal degeneration (axonopathy), reactive astrogliosis and, tau pathology, as shown by Bielschowsky silver staining **(A)** to disruption of axons and the presence of axonal swellings and spheroids; and by immunofluorescence (IF) for AT8 **(B)** & immunoblotting for AT180, AT8 and Tau5 (total tau) **(C)** to abnormal tau phosphorylation in neocortex. Scale bar, 50 µm. Data expressed as mean±SEM (two-way ANOVA with Bonferroni's correction and one-way ANOVA with Dunnett's correction). NS: not significant. *P < 0.05 and **P < 0.01.

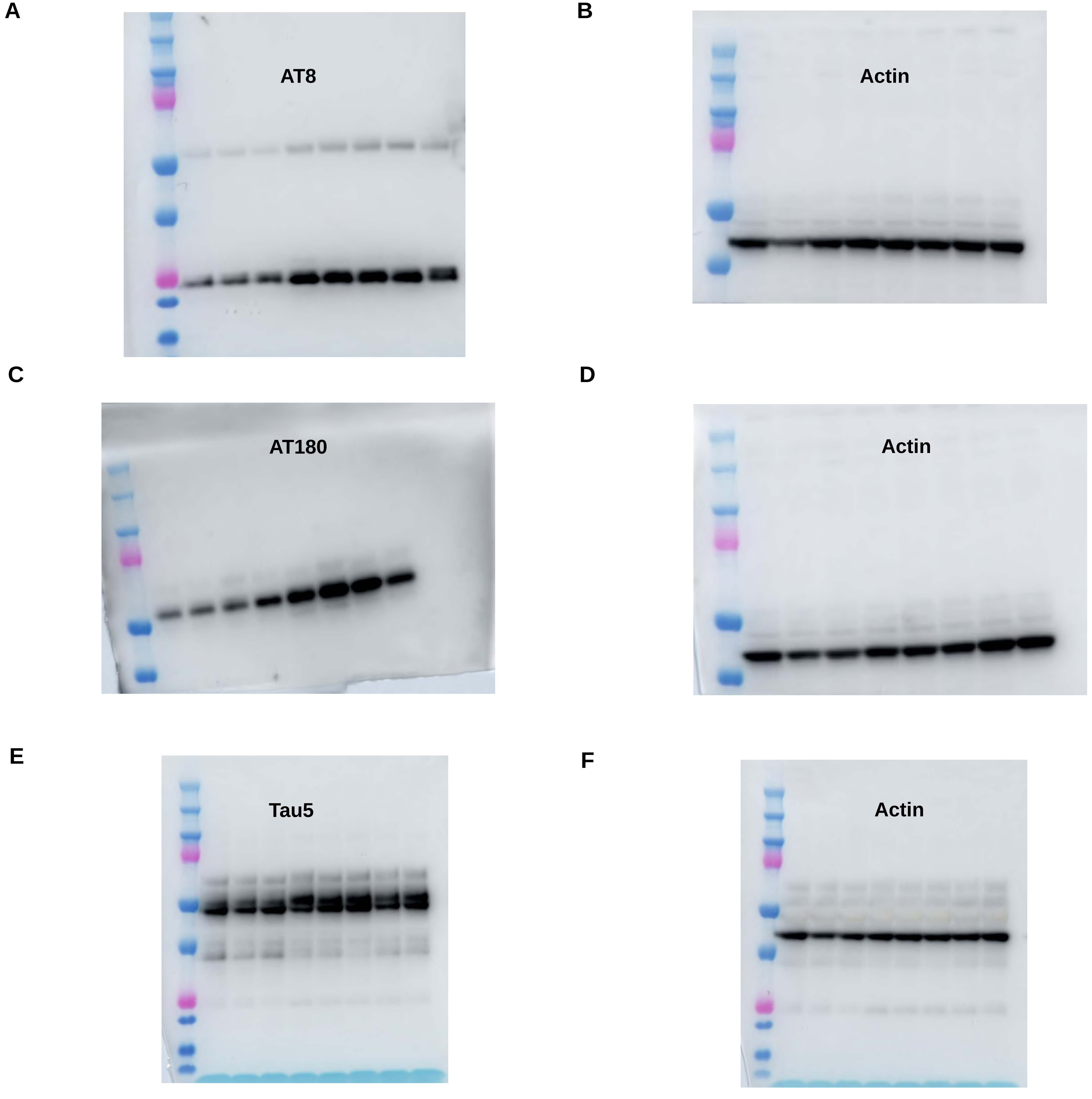

Supplementary Figure S3. Uncropped Western blot images. Uncropped western blot images for Fig. S2D **(A-F)**.

Supp. Figure 3

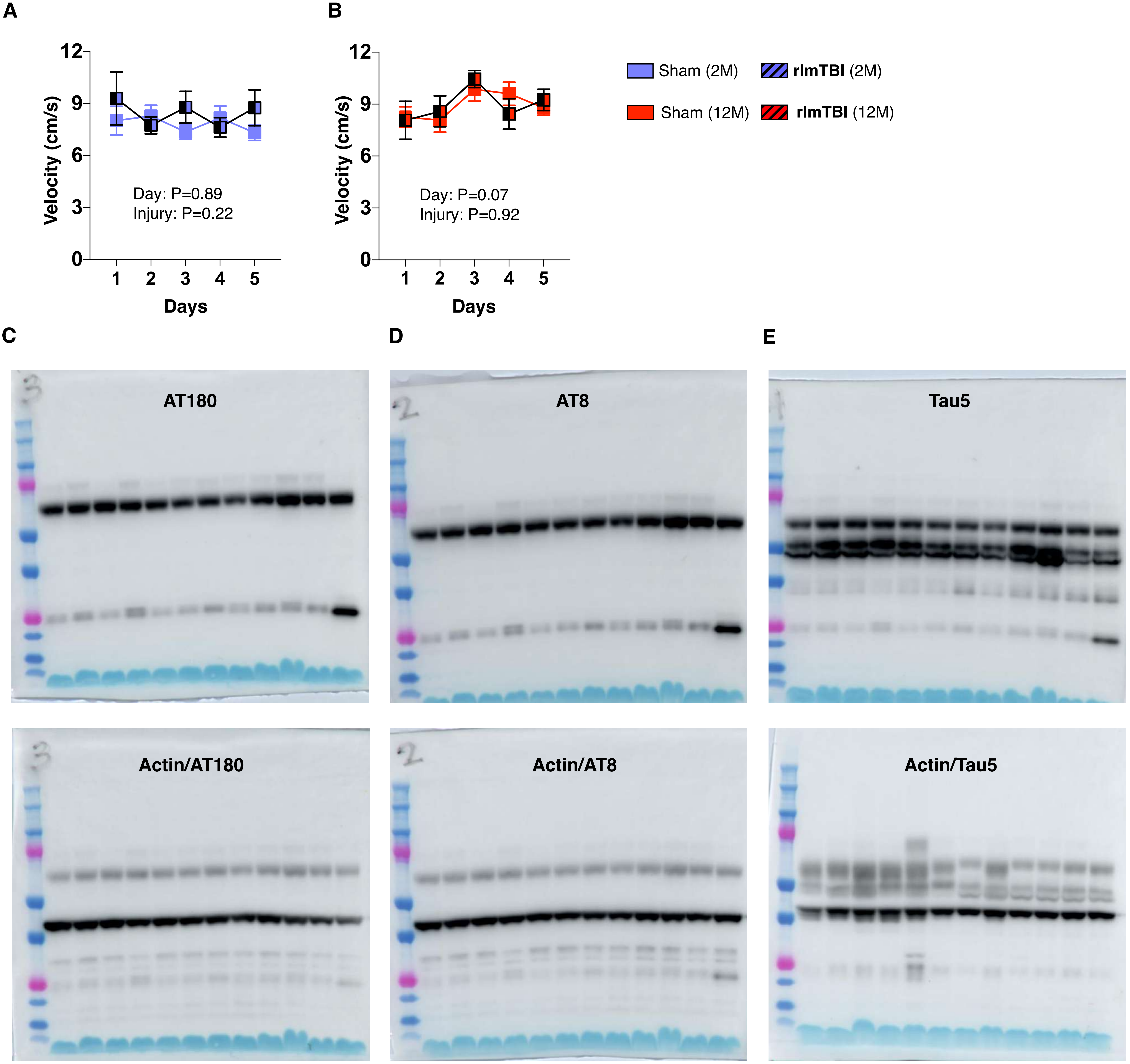

Supplementary Figure S4. The velocity of movement of 2M **(A)** and 12M **(B)** mice until the mice entered the escape tunnel during test are shown. **Uncropped Western blot images**. Uncropped western blot images for Fig. 1L **(C-E)**. Data expressed as mean±SEM (two-way ANOVA with Bonferroni's correction).

Supp. Figure 4

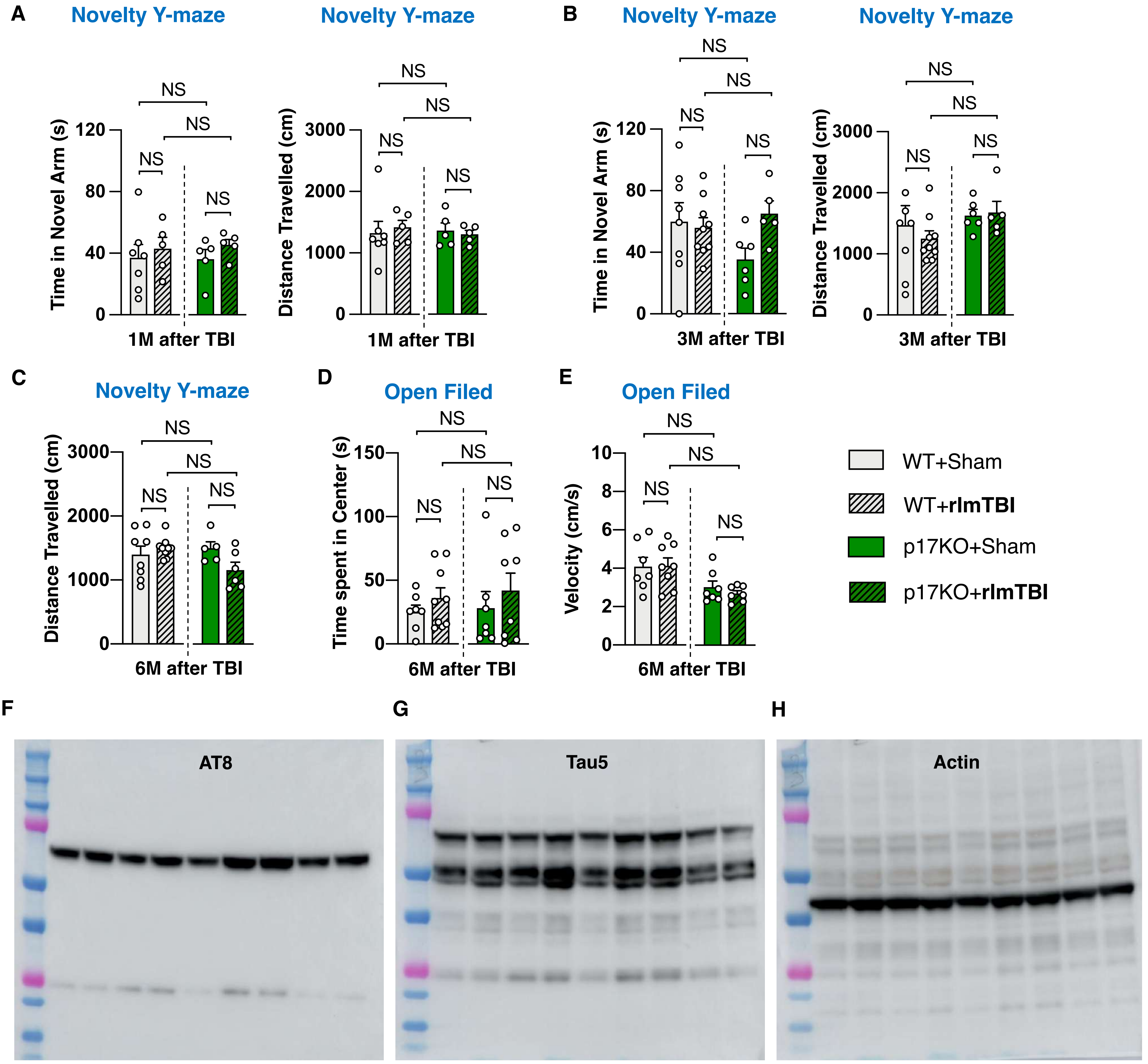

Supplementary Figure S5. Two-month-old male p17KO and C57BL/6J WT mice underwent rlmTBIs or sham injuries and followed by functional for 1, 3, and 6 months. **(A-C)** The Time spent in Novel arm and Distance travelled of the Y-maze test are shown at 1, 3, and 6 months after the last injury. Anxiety-like behavior assessed by bright-light open field in both groups, **(D)** time spent in center and **(E)** velocity at 6 months after the last injury. **Uncropped Western blot images**. Uncropped western blot images for Fig. 2P **(F-H)**. Data expressed as mean±SEM (two-way ANOVA with Bonferroni's correction).

Supp. Figure 5

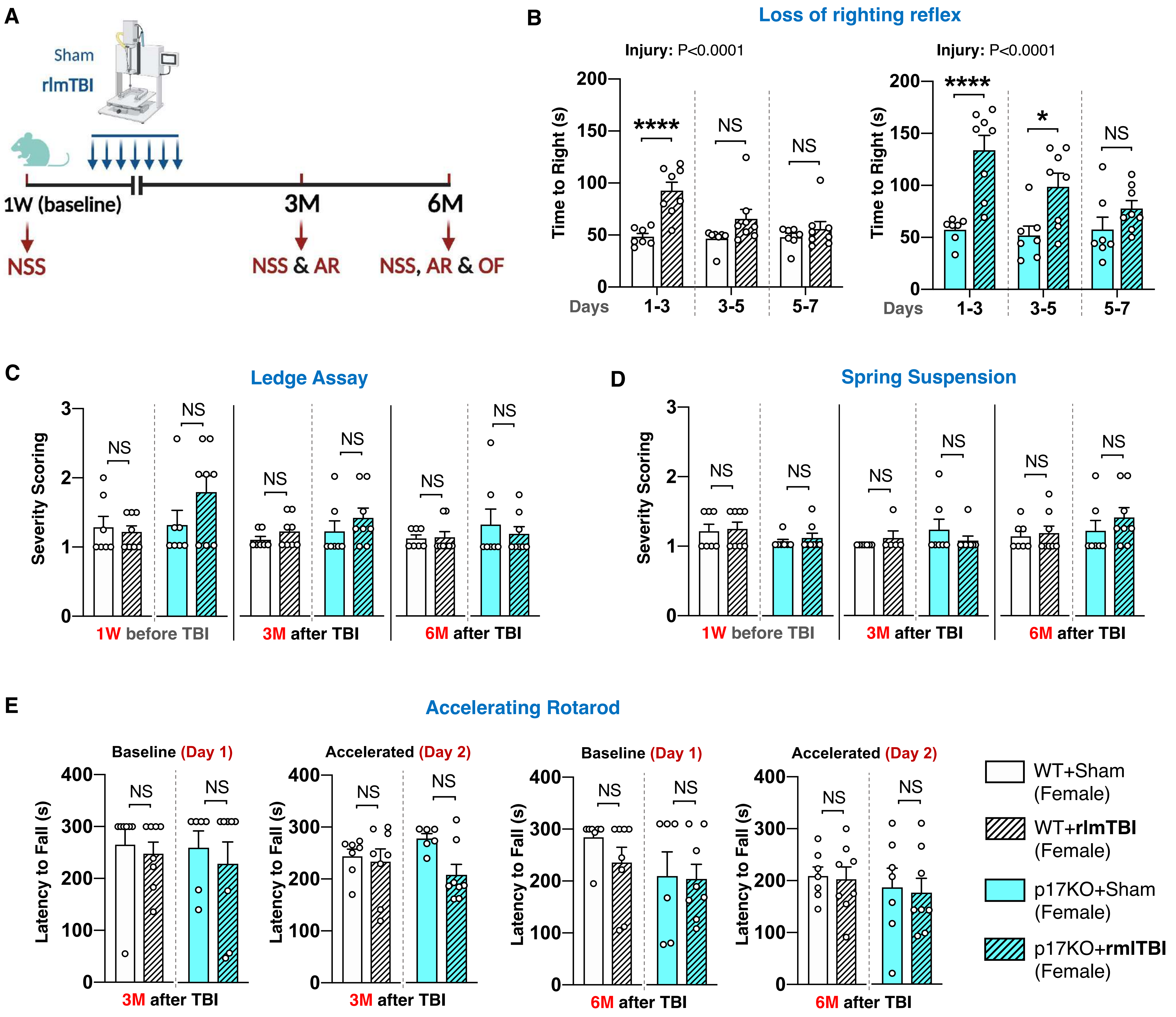

Supplementary Figure S6. (A) Experimental setup. Two-month-old female p17KO and C57BL/6J WT mice underwent rlmTBIs or sham injuries, and **(B)** the latency of their righting reflex was recorded, followed by functional and pathological examination for 6 months. Their neurological scoring (NSS) was longitudinally assessed by **(C)** Ledge Assay and **(D)** String Suspension test in mice before TBI, and 3, and 6 months after the last injury. **(E)** Sensorimotor competency was longitudinally assessed with the Accelerated Rotarod (AR) in mice 3 and 6 months after the last injury. Data expressed as mean±SEM (two-way ANOVA with Bonferroni's correction). NS: not significant. ****P < 0.0001.

Supp. Figure 6

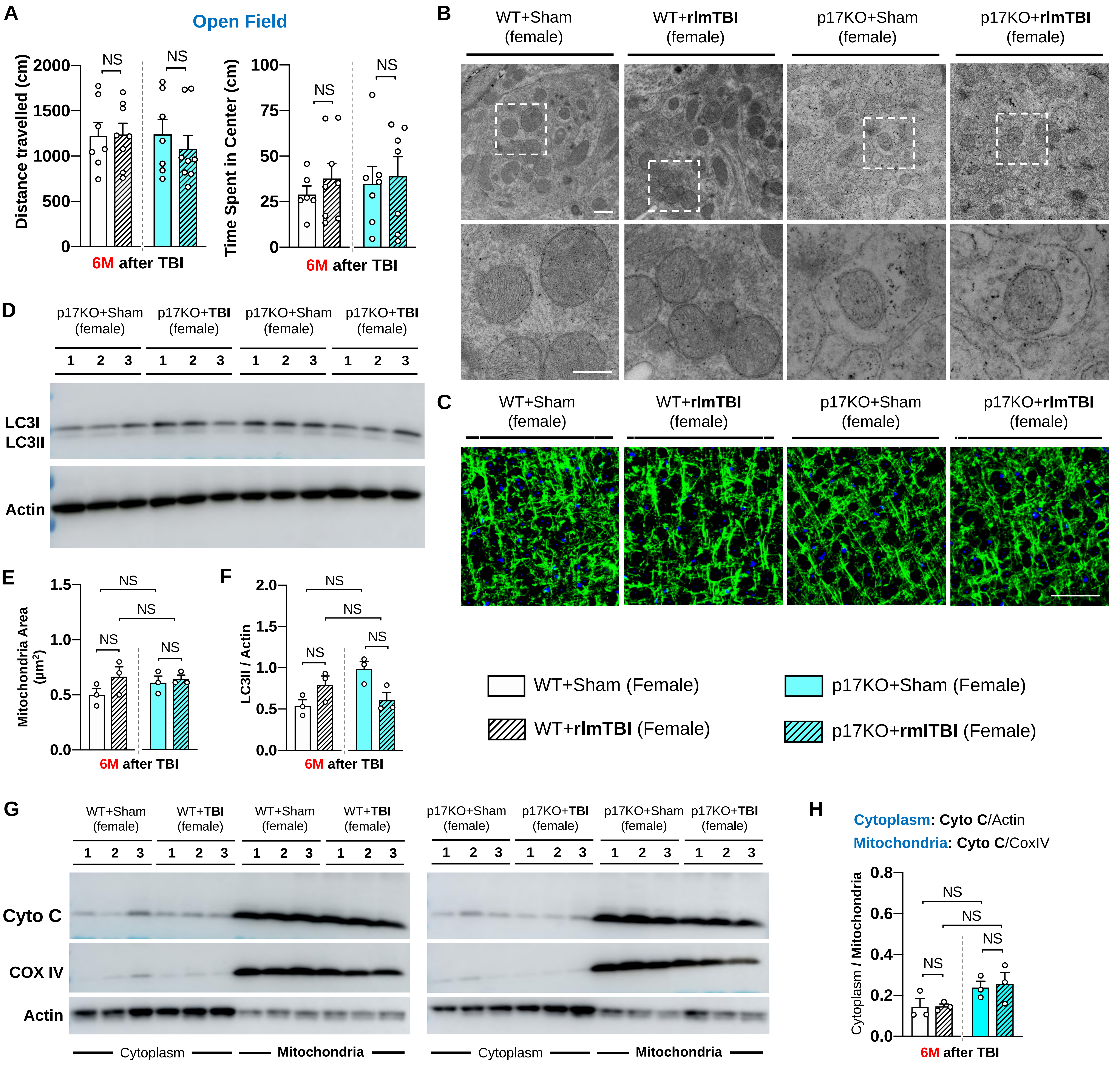

Supplementary Figure S7. (A) Anxiety-like behavior assessed by bright-light open field (OF) in both groups, distance travelled (left) and time spent in center (right) at 6 months after the last injury. The microstructural organization of myelinated axons and axonal integrity were assessed by immunostaining for **(B)** electron microscopy (scale bars, 1 μm) and **(C)** MBP (scale bar, 50 μm) in neocortex 6 months after the last injury. Inset images are high magnifications of representative areas. Bar graph showing the quantification of **(E)** average area of the mitochondria from electron microscopy in the neocortex of mice each group (n=3-5). **(D, F)** Whole-cell lysates were analyzed for lipidated LC3 (LC3-II) by immunoblotting in neocortex. **(G, H)** Mitochondrial and cytosolic Cytochrome c protein levels were analyzed with immunoblotting in neocortex with anti-Cytochrome c antibody. Loading standards were actin for homogenate and Cox IV for mitochondria (n=3-5). Data expressed as mean±SEM (two-way ANOVA with Bonferroni's correction). NS: not significant.

Supp. Figure 7

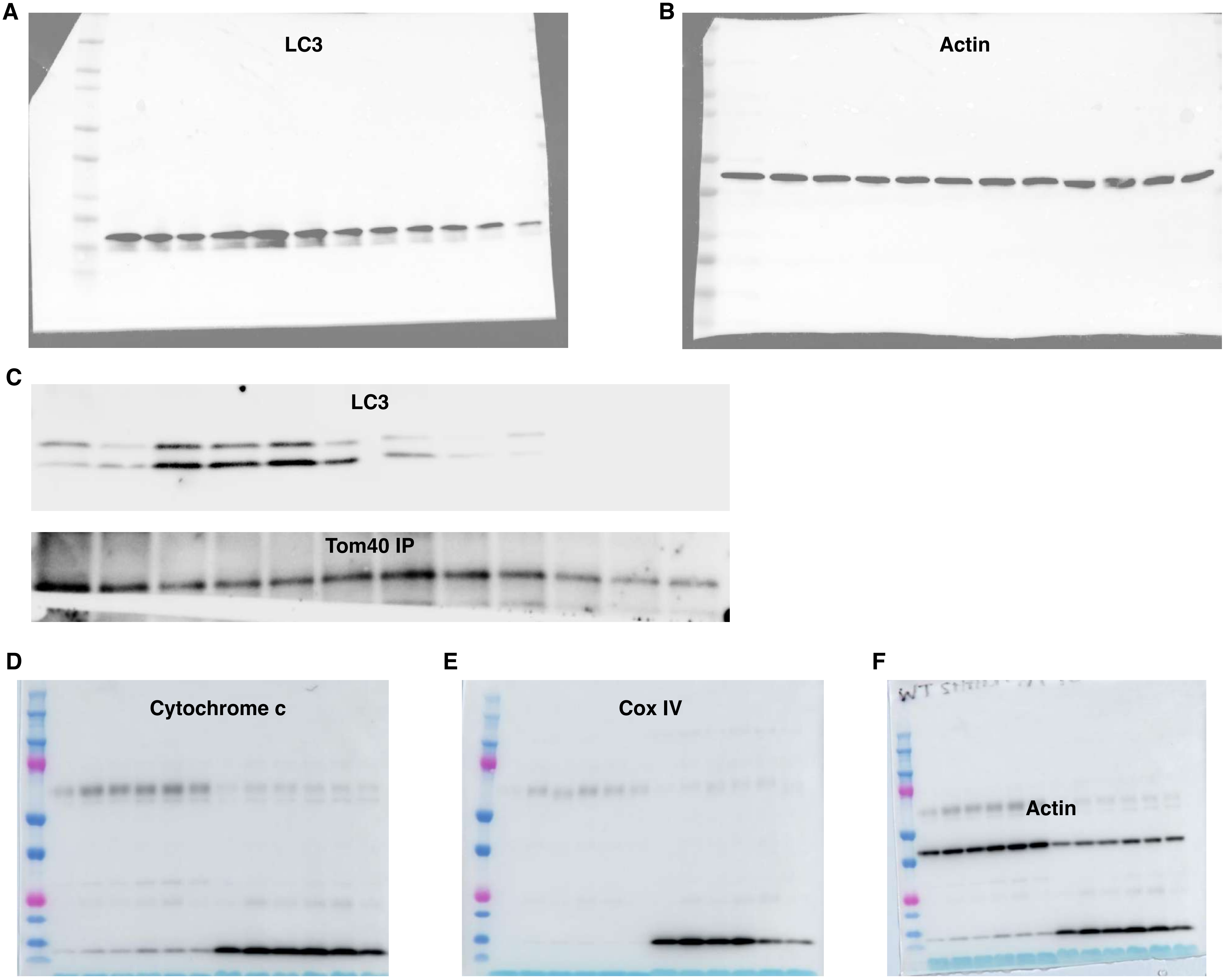

Supplementary Figure S8. Uncropped Western blot images. Uncropped western blot images for Fig. 3B **(A-B);** Fig. 3C **(C);** Fig. 3I **(D-E)** .

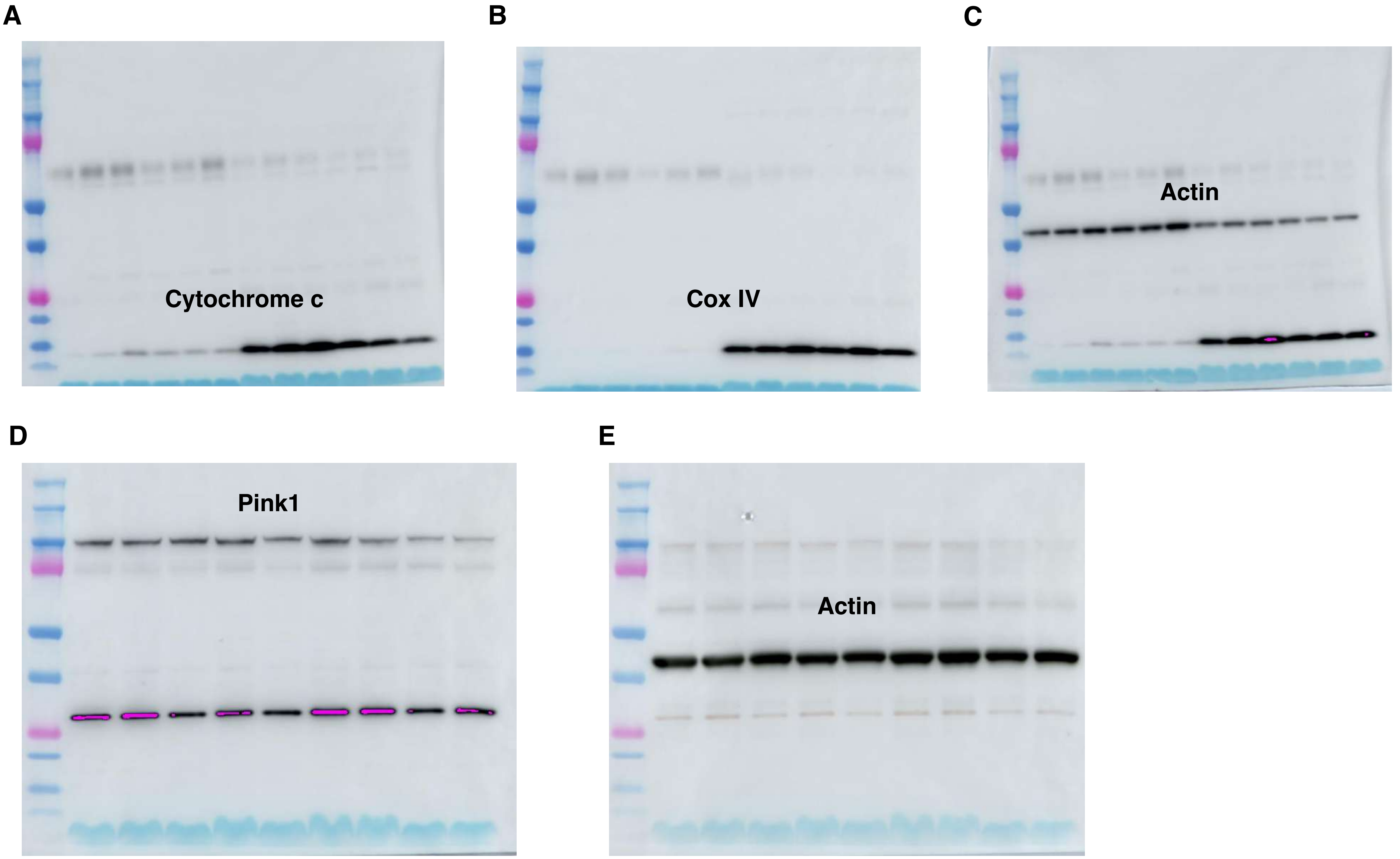

Supplementary Figure S9. Uncropped Western blot images. Uncropped western blot images for Fig. 3I **(A-C);** Fig. 3L **(D, E).**

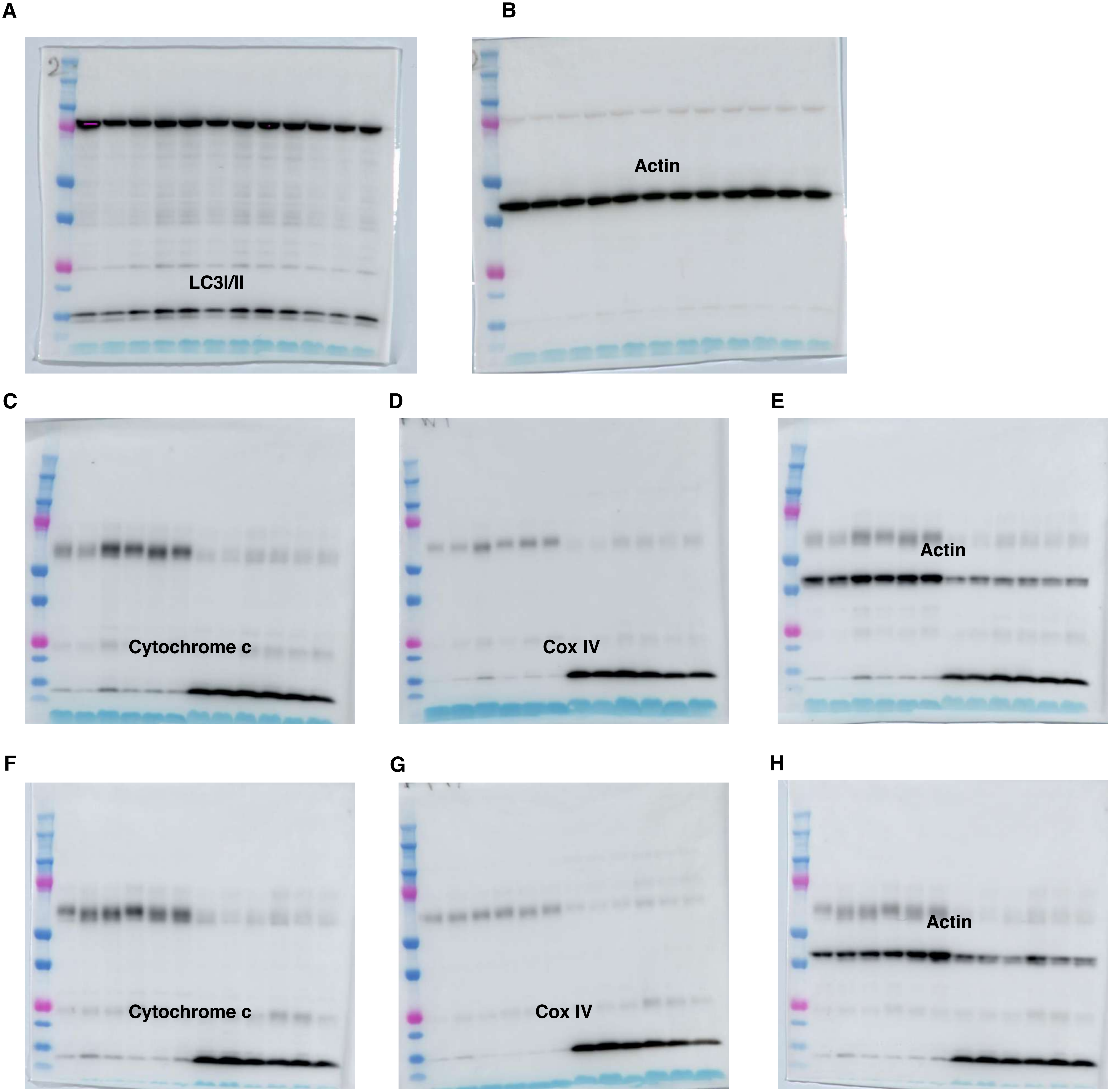

Supplementary Figure S10. Uncropped Western blot images. Uncropped western blot images for Fig. S7D **(A-B);** Fig. S7G **(C-H).**

Supp. Figure 10

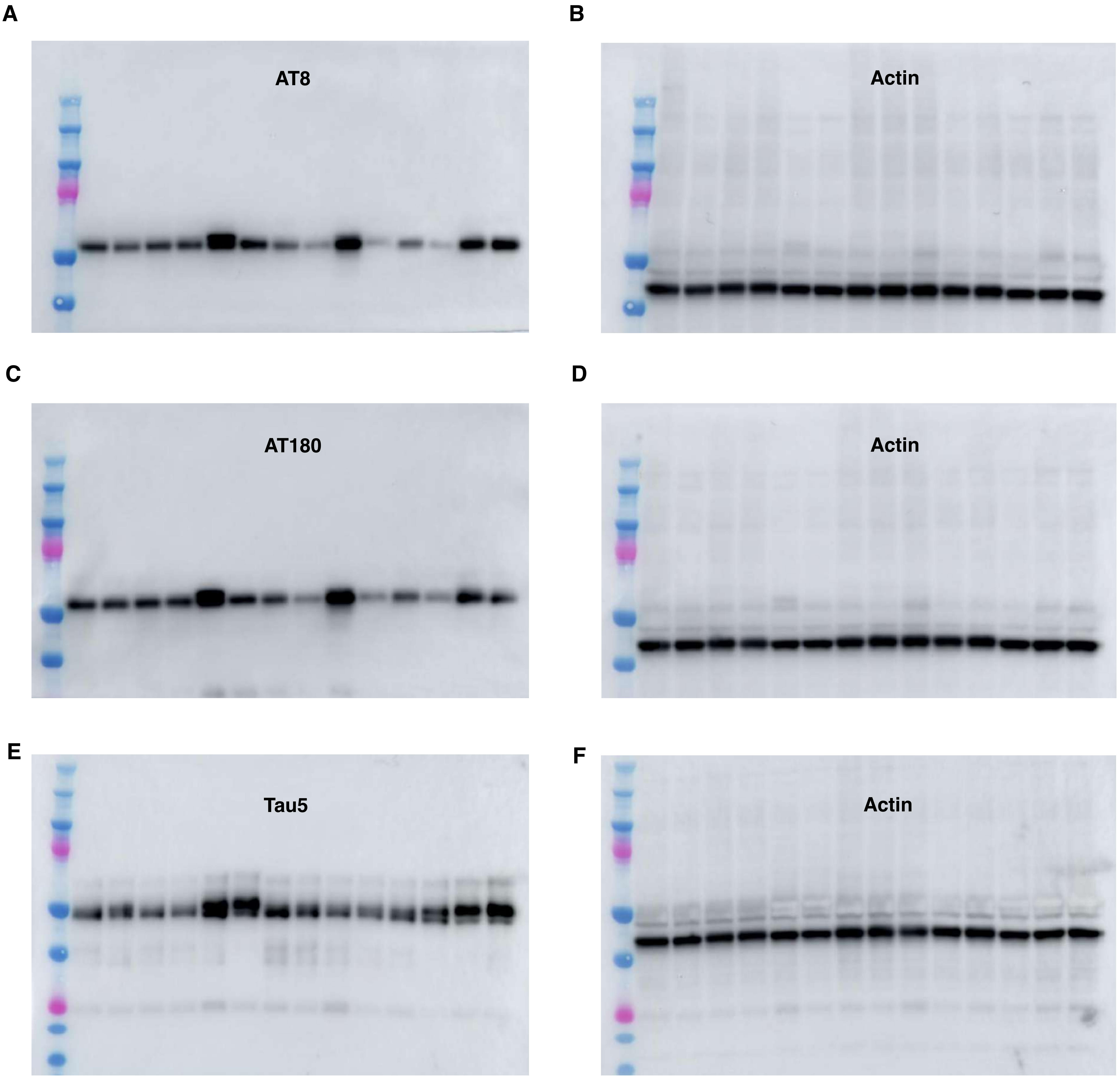

Supplementary Figure S11. Uncropped Western blot images. Uncropped western blot images for Fig. 4J **(A-F).**

Supp. Figure 11

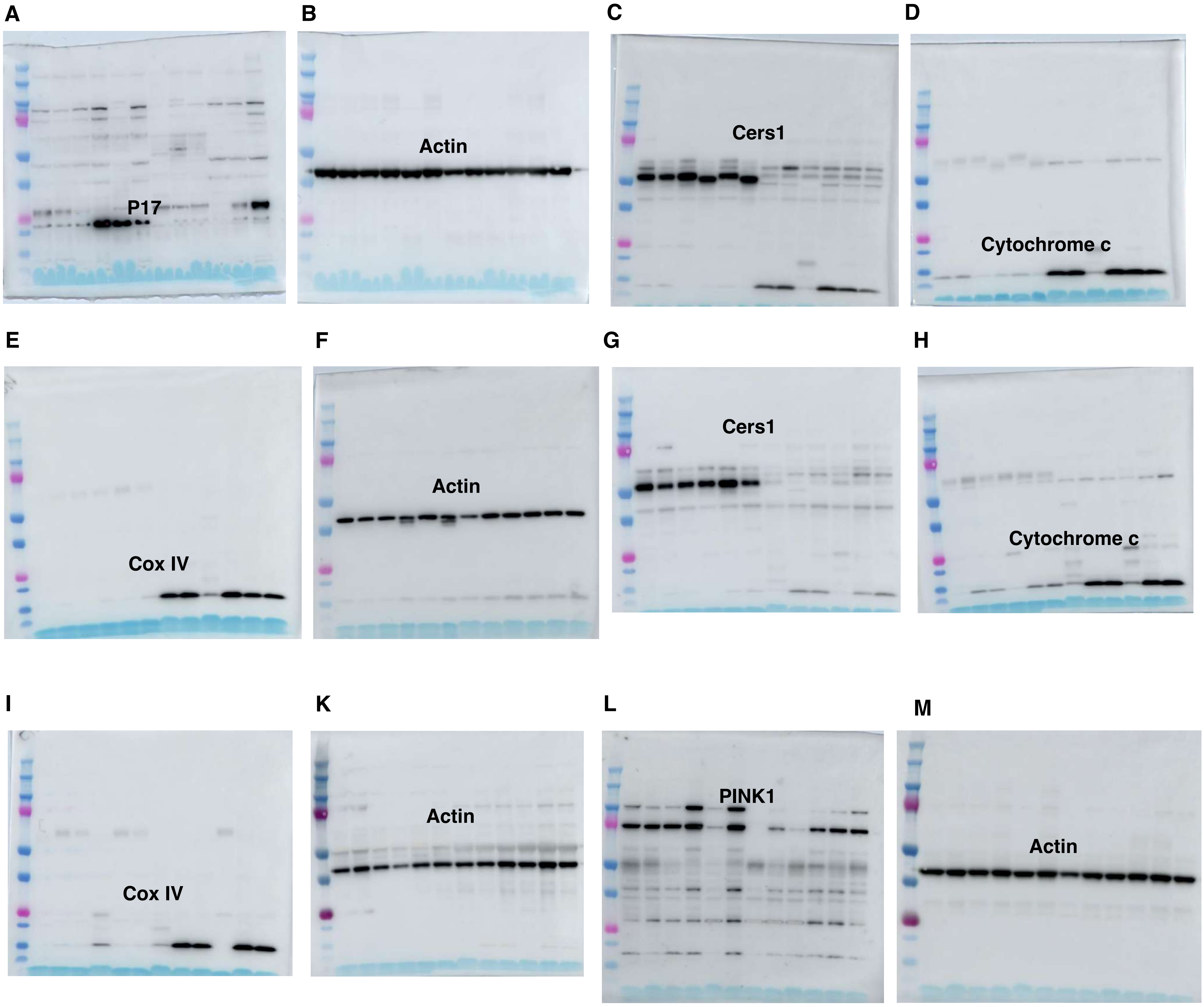

Supplementary Figure S12. Uncropped Western blot images. Uncropped western blot images for Fig. 5B **(A-B);** Fig. 5E **(C-H);** Fig. 5B **(L-M).**

Supp. Figure 12

Supplementary Table 1. The clinical information of patients with CTE and Control

	ID	gender	age	Primary diagnosis	Secondary diagnosis
**Control**	Control-1	Male	75	No-brain disease	-
	Control-2	Male	69	No-brain disease	-
	Control-3	Male	62	No-brain disease	-
	Control-4	Male	50	No-brain disease	-
	Control-5	Male	49	No-brain disease	-
	Control-6	Male	56	No-brain disease	-
**CTE**	CTE-1	Male	75	CTE	-
	CTE-2	Male	73	CTE	-
	CTE-3	Male	73	CTE	-
	CTE-4	Male	65	CTE	-
	CTE-5	Male	60	CTE	-
	CTE-6	Male	49	CTE	-
